# Recruitment of phospholipase Cγ1 to the non-structural membrane protein pK15 of Kaposi Sarcoma-associated herpesvirus promotes its Src-dependent phosphorylation

**DOI:** 10.1371/journal.ppat.1009635

**Published:** 2021-06-18

**Authors:** Naira Samarina, George Ssebyatika, Tanvi Tikla, Ja-Yun Waldmann, Bizunesh Abere, Vittoria Nanna, Michelangelo Marasco, Teresa Carlomagno, Thomas Krey, Thomas F. Schulz

**Affiliations:** 1 Institute of Virology, Hannover Medical School, Hannover, Germany; 2 German Center for Infection Research, Hannover Braunschweig Site, Hannover, Germany; 3 Institute of Organic Chemistry, Leibniz University Hannover, Hannover, Germany; 4 Excellence Cluster 2155 RESIST, Hannover Medical School, Hannover, Germany; 5 Centre for Structural Systems Biology (CSSB), Hamburg, Germany; University of North Carolina at Chapel Hill, UNITED STATES

## Abstract

Kaposi Sarcoma-associated herpesvirus (KSHV) causes three human malignancies, Kaposi Sarcoma (KS), Primary Effusion Lymphoma (PEL) and the plasma cell variant of multicentric Castleman’s Disease (MCD), as well as an inflammatory cytokine syndrome (KICS). Its non-structural membrane protein, pK15, is among a limited set of viral proteins expressed in KSHV-infected KS tumor cells. Following its phosphorylation by Src family tyrosine kinases, pK15 recruits phospholipase C gamma 1 (PLCγ1) to activate downstream signaling cascades such as the MEK/ERK, NFkB and PI3K pathway, and thereby contributes to the increased proliferation and migration as well as the spindle cell morphology of KSHV-infected endothelial cells. Here, we show that a phosphorylated Y^481^EEVL motif in pK15 preferentially binds into the PLCγ1 C-terminal SH2 domain (cSH2), which is involved in conformational changes occurring during the activation of PLCγ1 by receptor tyrosine kinases. We determined the crystal structure of a pK15 12mer peptide containing the phosphorylated pK15 Y^481^EEVL motif in complex with a shortened PLCγ1 tandem SH2 (tSH2) domain. This structure demonstrates that the pK15 peptide binds to the PLCγ1 cSH2 domain in a position that is normally occupied by the linker region connecting the PLCγ1 cSH2 and SH3 domains. We also show that longer pK15 peptides containing the phosphorylated pK15 Y^481^EEVL motif can increase the Src-mediated phosphorylation of the PLCγ1 tSH2 region *in vitro*. This pK15-induced increase in Src-mediated phosphorylation of PLCγ1 can be inhibited with the small pK15-derived peptide which occupies the PLCγ1 cSH2 domain. Our findings thus suggest that pK15 may act as a scaffold protein to promote PLCγ1 activation in a manner similar to the cellular scaffold protein SLP-76, which has been shown to promote PLCγ1 activation in the context of T-cell receptor signaling. Reminiscent of its positional homologue in Epstein-Barr Virus, LMP2A, pK15 may therefore mimic aspects of antigen-receptor signaling. Our findings also suggest that it may be possible to inhibit the recruitment and activation of PLCγ1 pharmacologically.

## Author summary

Kaposi’s Sarcoma-Associated Herpesvirus (KSHV) causes three human malignancies (Kaposi Sarcoma, Primary Effusion Lymphoma, Multicentric Castleman’s Disease) and an inflammatory condition, KICS. One of its non-structural membrane proteins, pK15, is expressed in tumor cells and has previously been shown to contribute to its ability to reactivate from latency and to its pathogenetic properties in endothelial cells by recruiting the cellular signaling enzyme phospholipase Cγ1 (PLCγ1). Here we investigate the interaction of pK15 with PLCγ1, report the structure of a PLCγ1 domain in complex with a pK15 peptide and show that pK15 primes PLCγ1 for phosphorylation by the cellular kinase Src. We also show that the pK15-dependent activation of PLCγ1 can be inhibited with a small peptide. Our findings therefore identify the pK15-PLCγ1 interaction as a putative druggable target and provide the basis for the development of small molecule inhibitors that could perhaps serve to inhibit KSHV replication and pathogenesis.

## Introduction

Kaposi’s sarcoma associated herpesvirus (KSHV) or Human Herpesvirus 8 (HHV-8), an oncogenic herpesvirus, is associated with three human malignancies, Kaposi’s sarcoma (KS), primary effusion lymphoma (PEL) and the plasma cell variant of multicentric Castleman’s disease (MCD), as well as with KSHV-associated inflammatory cytokine syndrome (KICS) [1–4]. Kaposi’s sarcoma, which was first reported in its ‘classical’ variant in 1872, is characterized by highly vascularized lesions with KSHV-infected, atypically differentiated endothelial spindle cells, abnormal blood vessels and red blood cells in the extracellular spaces [5,6]. The disease can be very aggressive, especially in AIDS patients [7,8].

Two phases of a life cycle–latent and lytic–are a characteristic feature of all herpesviruses, including KSHV. Shortly after KSHV entry, the virus initially expresses lytic genes, but then quickly establishes latency, which is characterized by the expression of very few viral genes [9–11]. Upon certain stimuli such as co-infection with other viruses, activation of Toll-like receptors, stimulation with cytokines, hypoxia, production of reactive oxygen species, KSHV reactivation from latency occurs and leads to the production of infectious viral particles [12–21]. While certain latent viral proteins such as LANA, vFLIP and vCyc, as well as a set of viral miRNAs are essential for the oncogenic properties of KSHV, the viral lytic phase is also thought to play an important role in the development of KS, MCD, PEL and KICS [9].

**Fig 1:**
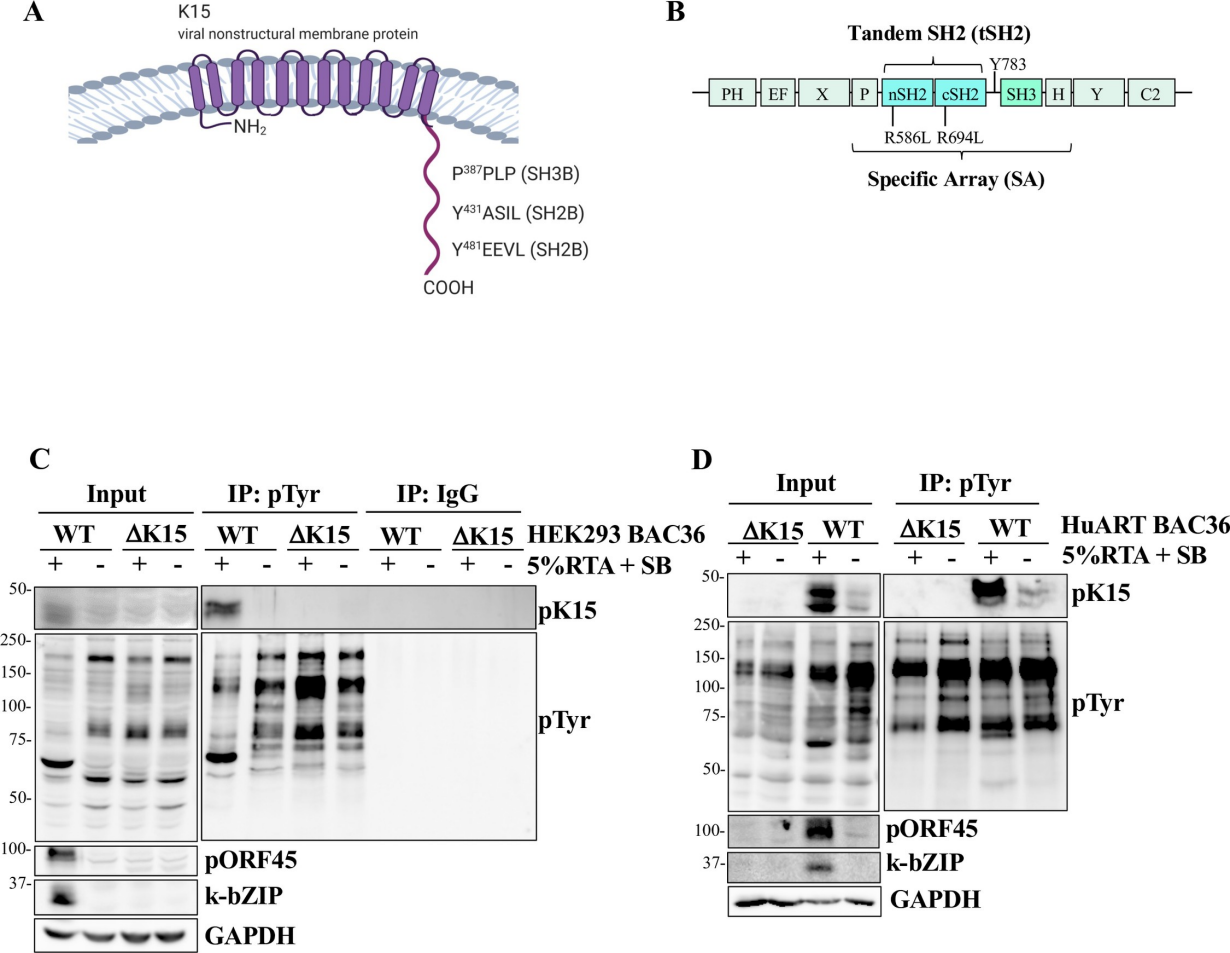
Full length K15 is phosphorylated in KSHV infected cells. (A) Diagram of the non-structural KSHV membrane protein pK15. Regions in its C-terminal domain discussed in this manuscript are indicated. (B) Diagram of PLCγ1. The position of the γ -specific array (SA), the tandem SH2 domain (tSH2) and the individual SH2 domains is shown. R^586^L and R^694^L refer to mutants in the nSH2 or cSH2 domain, Y^783^ to the tyrosine residue in the cSH2-SH3 linker segment. (C) HEK-293 stably transfected with BAC36 KSHV WT or BAC36 KSHVΔK15 were seeded in tissue culture flasks, followed after 24 hours by activating the lytic cycle by adding 5% tissue culture supernatant from insect cells expressing KSHV RTA and Na-butyrate (SB) (+); control cultures were not activated (-). Cells were lysed 48h after reactivation and immunoprecipitated with an antibody to pTyr or control IgG. (D) HuAR2T cells that had been stably infected with BAC36 KSHV WT or BAC36 KSHVΔK15 virus (produced in HEK293 BAC36 KSHV WT or BAC36 KSHVΔK15 cells) were plated in a 15 cm dish. After 48h the lytic cycle was induced as in (C) and 48h after reactivation cells were lysed and immunoprecipitated as in panel C.

The KSHV open reading frame (ORF) K15 is transcribed into a set of multiple alternatively spliced viral mRNAs. The longest splice variant encodes a 45 kDa protein, pK15, with predicted 12 hydrophobic membrane-spanning domains, as well as a short N-terminal and a longer C-terminal hydrophilic cytoplasmic tail [22,23]. The C-terminal cytoplasmic tail contains two Src homology 2 binding (SH2-B) sites and a Src homology 3 binding (SH3-B) (Fig 1A) that are important for the interaction with other proteins [22,24,25]. In KSHV-infected cells, the transmembrane protein pK15 is located on cytoplasmic membranes, in the perinuclear region as well as in the cell periphery, in the form of patches or punctate structures [22,24,26–30]. pK15 has been reported to be associated with cholesterol- and sphingolipid-rich membrane microdomains (‘lipid rafts’) [28,31]. It is expressed at low levels during latency, and its expression increases upon the induction of the lytic cycle, consistent with the fact that the major lytic switch protein, replication and transcription activator (RTA), can activate the K15 promoter [22,27,32,33]. Of note, pK15 was detected in tumour biopsies derived from PEL and KS patients at the mRNA and protein level [26,28,34]. By immunohistochemistry, the number of pK15-expressing endothelial spindle cells in some KS biopsies is comparable to the number of cells expressing the major latency protein LANA [28].

pK15 is known to activate several intracellular signaling cascades: it interacts with the tumor necrosis factor receptor-associated factors (TRAFs) TRAF-1,-2, and -3, activates PLCγ1-dependent signaling, the NF-κB pathway, as well as the two MAPK pathways MEK/ERK and SAPK/JNK and the downstream AP1 transcription factor [22,30,31,35–37]. In addition, pK15 upregulates the expression of cellular genes involved in inflammation and angiogenesis, among them DSCR1/RCAN1 (Down-syndrome critical region 1), which is expressed downstream of the PLCγ1—NFAT pathway [27,30,38]. pK15 induces constitutive PLCγ1 phosphorylation on tyrosine 783 and thereby its activation by binding directly to this protein [29,38]. The pK15 SH3-B as well as the distal SH2-B (Y481EEV) sites are important for this interaction. By activating PLCγ1-dependent cellular genes, K15 induces invasiveness and angiogenesis, which are the hallmarks of KS and it also plays role in KSHV lytic reactivation [28,29,38].

PLCγ1 is a member of the phospholipase C family, which includes several enzymes that hydrolyze phosphatidylinositol 4,5-biphosphate (PIP2) to two important second messengers, inositol 1,4,5-triphosphate (IP3) and diacylglycerol (DAG). DAG activates protein kinase C (PKC), whereas IP3 leads to the intracellular Ca2+ mobilization from the endoplasmic reticulum, which in turn activates calcineurin and leads to the translocation of the NFAT transcription factor to the nucleus [39–44]. PLCγ1 consists of two central catalytic domains forming the triose phosphate isomerase (TIM) α/β barrel (X- Y- box) and non-catalytic domains–a pleckstrin homology (PH), EF-hand and a C2 domain. The two catalytic domains are separated from each other by the specific array (SA), which consists of a split PH (spPH) domain that is separated by two SH2 domains (nSH2, cSH2), an SH3 domain and an intervening linker that contains a crucial tyrosine residue, Y^783^ (Fig 1B) [43–46].

Physiological PLCγ1 activation occurs following its recruitment to receptor or non-receptor tyrosine kinases (RTKs, TKs), which in turn phosphorylate the PLCγ1 SA domain on tyrosine residue 783. Either the aminoterminal nSH2 domain, or the carboxyterminal cSH2 domain have been found to bind to an activated RTK [46,47]. Activation of PLCγ1 occurs due to the intramolecular binding of the phosphorylated Y^783^ to the cSH2 domain, which leads to conformational changes in the protein and the release of the cSH2 domain from the catalytic core of PLCγ1, thus rendering the latter accessible to the substrate PIP2 [43,44,46–49].

In contrast to cellular RTKs, pK15 is not a tyrosine kinase, and one of the unanswered questions therefore is how pK15 can activate the phosphorylation of PLCγ1. As a first step towards answering this question, we studied the interaction of pK15 with PLCγ1 in molecular detail. We identified regions of pK15 and PLCγ1 that are crucial for their interaction in cells as well as *in vitro*, and determined the crystal structure of the PLCγ1 tandem SH2 domain in complex with a phosphorylated peptide representing the C-terminal end of the pK15 cytoplasmic domain. Our results suggest that the interaction of a phosphorylated pK15 with PLCγ1 primes the latter for phosphorylation by src kinase. pK15 may therefore act as a ‘scaffold’ to promote the phosphorylation of PLCγ1 by cellular RTKs or TKs. We also show that it is possible to inhibit the pK15-dependent phosphorylation of PLCγ1 with a small pK15-derived peptide occupying the PLCγ1 cSH2 domain.

## Results

### pK15 phosphorylation in KSHV infected cells and its role in the interaction with PLCγ1

We and others have previously shown that the cytoplasmic tail of pK15 is phosphorylated on tyrosine residue Y^481^ in cells that were transfected with a K15 expression vector [24,27–29,31,38]. To show that pK15 phosphorylation takes place in KSHV infected cells, we immunoprecipitated all proteins phosphorylated on tyrosine from HEK293 cells carrying either a bacmid containing the wt KSHV genome (BAC36; WT) or a KSHV genome, in which the first seven exons of ORFK15 had been replaced with a kanamycin cassette (ΔK15) [38]. We were able to detect pK15 bands after phosphotyrosine immunoprecipitation in the HEK293 cells carrying the KSHV WT, but not KSHV ΔK15 virus, using an antibody to pK15 (Fig 1C, blot labelled ‘pK15’). The abundance of the phosphorylated pK15 is low, as we could not detect it by immunoblotting with an antibody to phosphotyrosines in either the input lysates, or after immunoprecipitation with phosphotyrosine antibody–coated beads (Fig 1C, blots labelled ‘pTyr’). Moreover, in HEK293 cells carrying the KSHV WT, pK15 could only be detected (Fig 1C, input), or immunoprecipitated with an anti-phosphotyrosine antibody (Fig 1C, IP:pTyr), upon the induction of the lytic cycle by RTA and sodium butyrate (indicated by the expression of the early lytic proteins k-bZIP and pORF45 in Fig 1C, input). We obtained a similar result in an immortalised endothelial cell line, HuAR2T, which had been stably infected with virus produced in HEK293 cells carrying either KSHV BAC 36 or KSHV BAC36 ΔK15 [28,38] (Fig 1D). In these endothelial cells, phosphorylated pK15 was already expressed weakly before the induction of the lytic cycle (Fig 1D, Input). This result indicates that pK15 is phosphorylated in KSHV-infected epithelial and endothelial cells, in particular following the reactivation of the lytic replication cycle.

pK15 interacts with many cellular proteins having diverse functions [22,26,29,31,38,50,51]. Among them, the interaction with phospholipase C gamma 1 (PLCγ1) has been shown to be responsible for the invasive and angiogenic phenotype of KSHV-infected endothelial cells as well as for KSHV reactivation from latency [28,29,38]. Considering that one of the SH2 binding sites of pK15 (Y^481^EEV; Fig 1A) is crucial for this interaction [38] and that pK15 is phosphorylated on the tyrosine residue within this motif [29,31,38], it was important to test the effect of pK15 phosphorylation on its binding to PLCγ1. For this, we phosphorylated the purified cytoplasmic domain of pK15 *in vitro* and measured its binding to transfected or purified recombinant PLCγ1 or PLCγ1 fragments.

**Fig 2:**
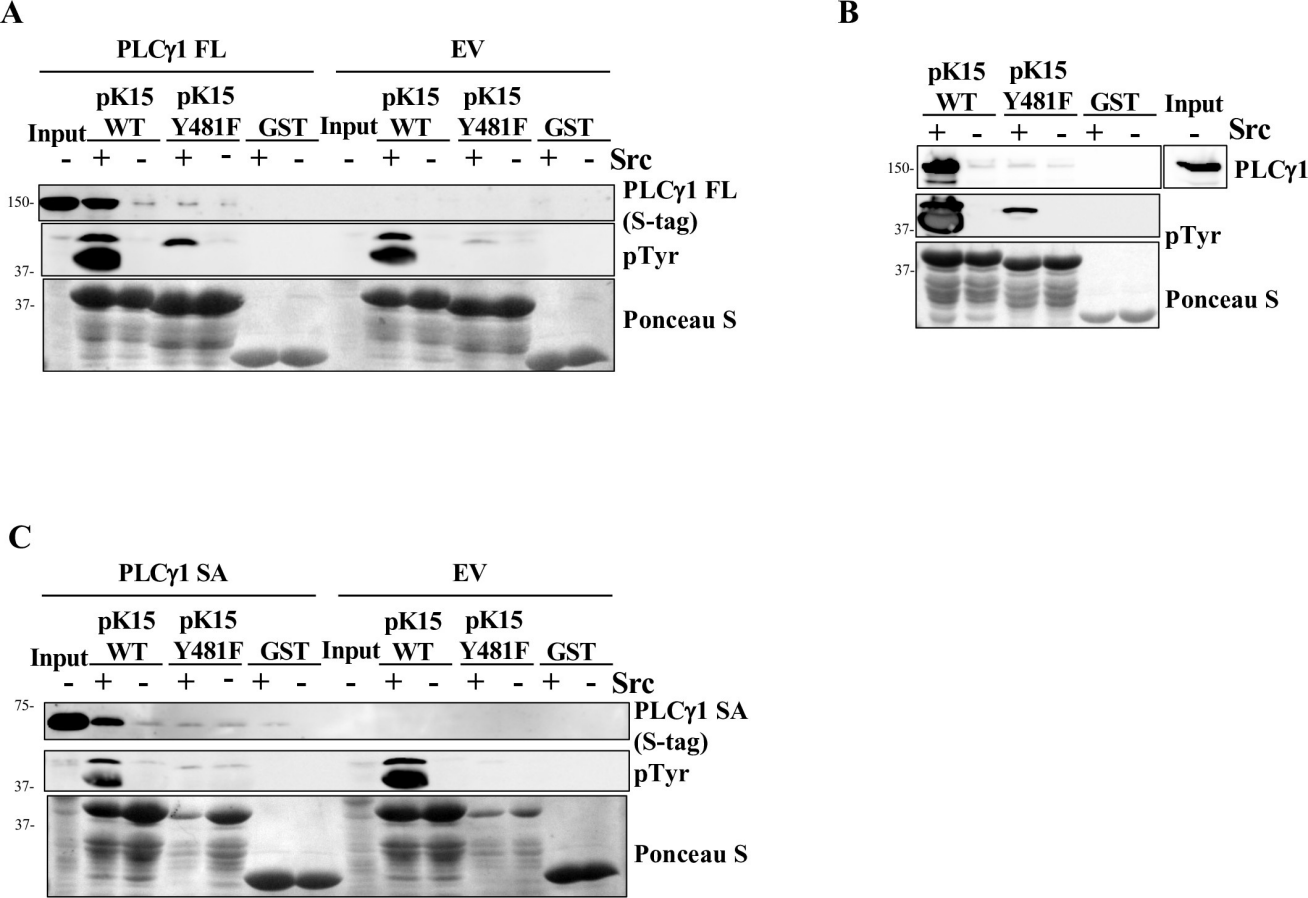
K15 phosphorylation results in a stronger binding to PLCγ1. (A) An expression plasmid for an S-tagged full length PLCγ1 or the empty vector (EV) were transfected in HEK-293T cells. After 30h cells were lysed and a GST-pulldown assay was performed with GST-fused pK15 CT (WT), the pK15 CT Y^481^F mutant (Y^481^F) or GST that had been bound to glutathione beads and previously phosphorylated by GST-6xHis Src (+) or left unphosphorylated (-). Bound proteins were analysed by WB using antibodies to the S tag on PLCγ1 and pTyr. (B) Untransfected HEK-293T cells were lysed and a GST-pulldown assay with pre-phosphorylated (+) or unphosphorylated (-) GST-fused pK15 CT WT/Y^481^F or GST was performed and analysed by WB as in (A). (C) An expression plasmid for an S-tagged PLCγ1 SA or the empty vector were transfected in HEK-293T cells. After 30h, cells were lysed and a GST-pulldown was performed as in panel A.

We expressed and purified the pK15 cytoplasmic tail (pK15 CT) fused to GST at its N-terminal end in bacteria and phosphorylated it *in vitro* using a commercially obtained human GST-6xHis Src, or a recombinant chicken 2xStrep Src kinase expressed in insect cells. In parallel, the GST-pK15 CT domain carrying the Y^481^F mutation, or GST protein alone, were subjected to the same procedure. Phosphorylation of pK15 CT^WT^ resulted in a dominant (bottom) and a minor (top) pK15 band that could be detected by Western blot using an antibody to phosphorylated tyrosine residues (Figs 2A–2C, middle panel labelled ‘pTyr’ and S1), while phosphorylation of pK15 CT^Y481F^ only produced the minor band (top). This minor band results from the phosphorylation of the second SH2-binding site (Y^431^ASL) in the pK15 CT domain, since submitting a GST-pK15 CT domain carrying a Y^431^F mutation to *in vitro* phosphorylation by recombinant Src kinase failed to produce this band (S1 Fig). Following in vitro phosphorylation, pK15 CT^WT^ and pK15 CT^Y481F^ were used to pull down transiently expressed (Fig 2A) or endogenous (Fig 2B) PLCγ1 from HEK 293 lysates. We found that, for both transfected and endogenous PLCγ1, *in vitro* phosphorylation of the cytoplasmic tail of pK15 CT^WT^ led to a substantial increase in its interaction with PLCγ1, while phosphorylation of the mutant pK15 CT^Y481F^ only showed a minimal interaction with PLCγ1 (Fig 2A and 2B).

The PLCγ1 specific array (SA), which is important for the interaction with pK15 [29,38], contains one SH3 and two SH2 domains, with the latter constituting the tandem SH2 (tSH2) domain (Fig 1B). As shown in Fig 2C, we also transfected the isolated PLCγ1 SA region into HEK 293 cells and measured its binding to the *in vitro* phosphorylated pK15. Similar to full length PLCγ1 (Fig 2A and 2B), phosphorylated pK15 CT^WT^ bound more strongly to the SA region than the phosphorylated pK15 CT^Y481F^ mutant; a marginal binding to PLCγ1 SA was observed without prior *in vitro* phosphorylation of pK15 CT^WT^ by Src kinase (Fig 2C).

### Role of the PLCγ1 SH2 domains in the interaction with phosphorylated pK15

Having shown that phosphorylation of the distal SH2-binding motif (Y^481^EEVL) in the pK15 cytoplasmic domain is required for its recruitment of PLCγ1, we next investigated the involvement of the two PLCγ1 SH2 domains in this interaction. In a first step, we compared the interaction of phosphorylated pK15 CT with transfected full-length PLCγ1, the PLCγ1 SA domain, the PLCγ1 tSH2 domain, or individual SH2 domains and with PLCγ1 mutants, in which arginine residues at positions 586 in the nSH2 domain and/or position 694 in the cSH2 domain had been substituted to leucines (Fig 1B). These arginine residues form a central part of the phospho-tyrosine binding pockets within the nSH2 or cSH2 domains, into which the phosphorylated pK15 would be expected to bind.

**Fig 3:**
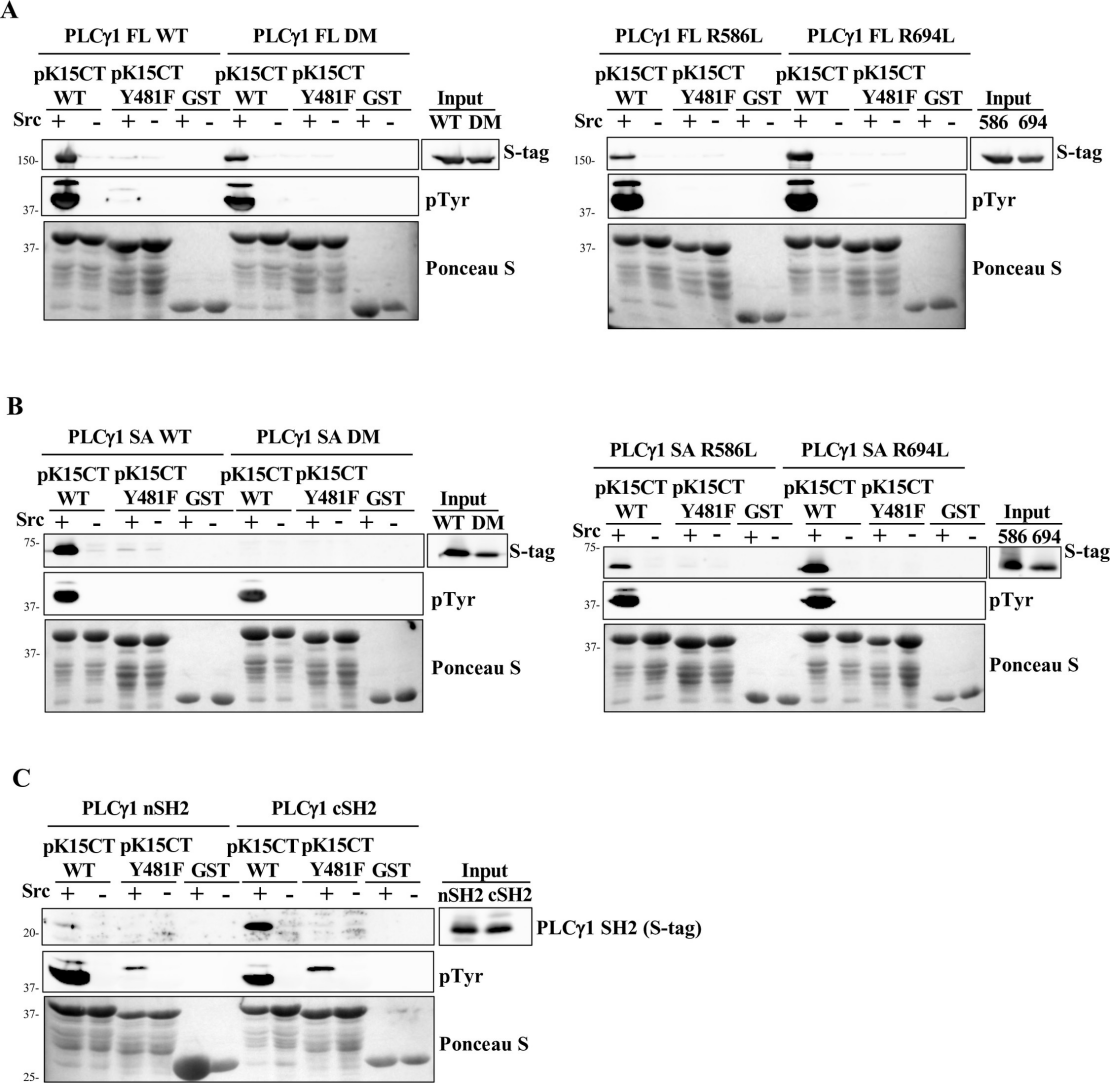
Contribution of individual PLCγ1 SH2 domains to the interaction with phosphorylated pK15. The pK15 CT fused to GST, its Y^481^F mutant, or GST alone were bound to glutathione beads, phosphorylated *in vitro* (+) or left unphosphorylated (-), and a GST pulldown was performed 32h after transfection into HEK-293T cells of expression vectors for S-tagged full-length PLCγ1 (FL) (A) or PLCγ1 SA (B) containing the indicated mutations. (C) pK15 CT WT, its Y^481^F mutant or GST were bound to glutathione beads, phosphorylated *in vitro* (+) or left unphosphorylated (-), and used for a GST pulldown assay 32h after transfecting HEK 293T cells with expression vectors for S-tagged individual PLCγ1 nSH2 or cSH2 domains.

GST pulldown assays with phosphorylated pK15 CT demonstrated that, when full-length PLCγ1 or the PLCγ1 SA domain were transiently expressed in HEK 293T cells, mutating either R586 or R694 in, respectively, the PLCγ1 nSH2 or cSH2 domains led to a moderately reduced binding to phosphorylated GST-pK15 CT in the case of full-length PLCγ1 WT (Fig 3A) and strongly reduced binding in the case of the PLCγ1 SA domain (Fig 3B). Mutating both SH2 domains simultaneously completely eliminated the binding of phosphorylated pK15 CT to the PLCγ1 SA domain, but not to full length PLCγ1 (Fig 3A and 3B), suggesting that other regions in PLCγ1 located outside the SA domain may also contribute to this interaction with *in vitro* phosphorylated pK15. We also investigated the individual contribution of the two PLCγ1 SH2 domains to the interaction with phosphorylated pK15 CT by transfecting expression vectors for the nSH2 or cSH2 domain into HEK 293 cells and performing a GST-pulldown assay with *in vitro* phosphorylated pK15 CT^WT^ or pK15 CT^Y481F^. In this experiment, the phosphorylated pK15 CT^WT^ bound preferentially to the PLCγ1 cSH2 domain compared to its nSH2 domain (Fig 3C).

**Fig 4:**
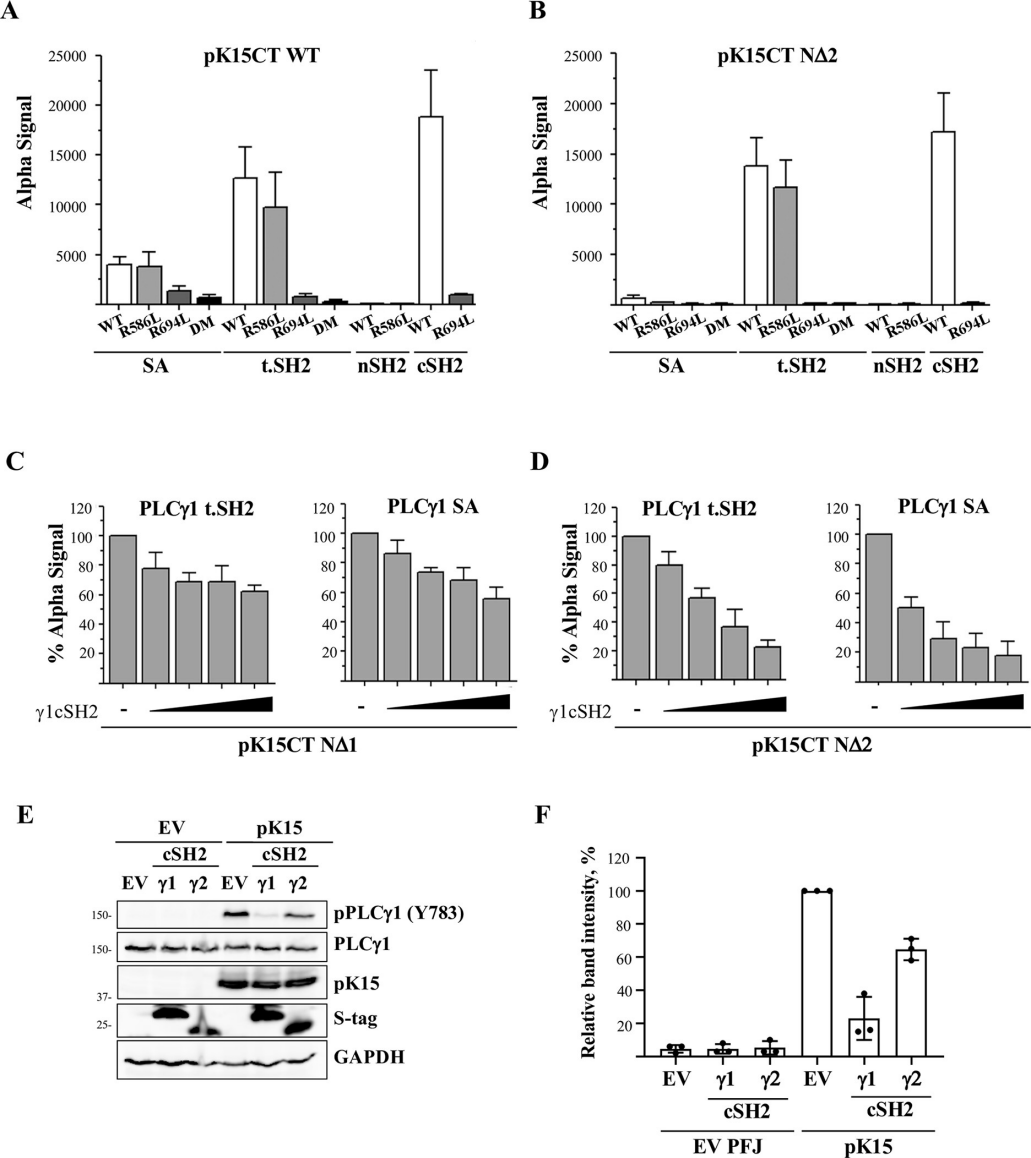
The PLCγ1 cSH2 domain is required for the binding of PLCγ1 to phosphorylated pK15 *in vitro* and may serve as a dominant-negative inhibitor. GST-fused pK15 cytoplasmic tail WT (A), or its truncated mutant N2 [51] (B), were phosphorylated *in vitro* by 2xStrep-Src and an AlphaLISA was performed with the indicated His-tagged fragments of PLCγ1. Mean and standard deviation from at least two independent experiments with two technical replicates each are depicted. GST fusion proteins of the N1 (C) or N2 (D) truncation mutants of the pK15 cytoplasmic tail [51] were phosphorylated *in vitro* by 2xStrep-Src and their interaction with recombinant purified PLCγ1 tSH2 (tandem SH2) or SA (γ-specific array) domains was measured by AlphaLISA in the presence of increasing concentrations (300 nM, 600 nM, 900 nM, 1200 nM) of the purified PLCγ1 cSH2. Mean and a standard deviation from at least three independent experiments with two technical replicates each are depicted.–: empty vector (pTriex4). (E) An expression vector (PFJ) expressing full length pK15 or empty PFJ (EV) were co-transfected in HEK-293T cells together with the isolated cSH2 domains of PLCγ1 or PLCγ2 or their empty vector pTriex4. After 48h lysates were collected and analysed by Western blot using an antibody to PLCγ1 pY^783^ to assess the level of PLCγ1 phosphorylation. (F) The intensities of the pPLCγ1 bands shown in panel E were quantified by densitometry, normalized to the values obtained with the empty vector (EV) in the presence of pK15, and plotted as relative values. The diagram shows the results obtained in three independent experiments.

We next quantified the contribution of the PLCγ1 γ-specific array (SA) and its subdomains (tSH2, nSH2 or cSH2) to the interaction with phosphorylated pK15 CT in an AlphaLisa experiment, in which the interaction of phosphorylated purified GST-pK15 CT with recombinant purified His-tagged PLCγ1 domains is measured by adding glutathione-coated donor beads and Ni-derivatised acceptor beads. As the two protein ligands interact, donor and acceptor beads are brought into close proximity and excitation of the donor beads at 680 nm generates a luminescence signal at 615 nm emanating from the acceptor beads. In this experiment, in vitro phosphorylated pK15 CT^WT^ bound more strongly to the PLCγ1 tSH2 domain than to the PLCγ1 SA domain; for both the SA and tSH2 domains the interaction was entirely dependent on the two SH2 domains, since the PLCγ1 R^586^L and R^694^L double mutant (DM) failed to interact with phosphorylated pK15 CT^WT^ (Fig 4A). In the context of both PLCγ1 SA and PLCγ1 tSH2, the nSH2 R^586^L mutant bound pK15 CT^WT^ to a similar extent as wt PLCγ1 SA and PLCγ1 tSH2, while mutating the cSH2 domain (R^694^L) markedly reduced binding (Fig 4A). In keeping with this observation, only the PLCγ1 cSH2 domain (and not the nSH2 domain) interacted with phosphorylated pK15 CT^WT^ when tested individually, and this interaction was abolished by the R^694^L mutation in the cSH2 domain (Fig 4A), thus suggesting that the PLCγ1 cSH2 domain is crucial for its binding to the phosphorylated pK15 CT domain.

An *in vitro* phosphorylated pK15 CT^N2^ only bound weakly to PLCγ1 SA, not to the PLCγ1 nSH2 domain, but did interact with the tSH2 and cSH2 domains (Fig 4B). This truncated pK15 CT^N2^ contains pK15 amino acids 410–489 fused to GST [51] and thus lacks the P^387^PLP SH3 binding site in the pK15 cytoplasmic domain (Fig 1A). The fact that *in vitro* phosphorylated pK15 CT^N2^ binds strongly to the PLCγ1 tSH2 domain but not to the PLCγ1 SA domain, while pK15 CT^WT^ does interact with the PLCγ1 SA, could suggest an involvement of the PLCγ1 SH3 domain in the interaction with pK15 CT^WT^. The stronger binding of pK15 CT^WT^ and pK15 CT^N2^ to the PLCγ1 tSH2 domain than to the longer PLCγ1 SA domain could be explained by the pseudo-cyclic conformation of the latter, whose N- and C-termini are known to be brought together in a non-covalent fashion [46]; this ‘closed’ conformation may be harder to access for pK15 CT and pK15 CT^N2^ than the tSH2 domain.

### The PLCγ1 cSH2 domain can serve as a dominant negative inhibitor of the K15-PLCγ1 interaction

We previously showed that, when transfected into cells, the isolated cSH2 domain of PLCγ2 can be used to inhibit the K15–PLCγ1 interaction and subsequent PLCγ1 activation in a dominant negative manner [29]. In view of the contribution of the PLCγ1 cSH2 domain to the interaction of phosphorylated pK15 CT with PLCγ1 (Figs 3, 4A and 4B) we therefore explored whether it might have a similar inhibitory effect as PLCγ2 cSH2. Using the AlphaLISA assay we could show that the purified PLCγ1 cSH2 domain can directly inhibit the interaction between two *in vitro* phosphorylated pK15 fragments, pK15 CT^N1^ or pK15 CT^N2^, and PLCγ1 SA or PLCγ1 tSH2 in a dose-dependent manner (Fig 4C and 4D). pK15 CT^N1^ contains pK15 amino acids 365–489 fused to GST [51] and therefore retains the P^387^PLP SH3 binding site in the pK15 cytoplasmic domain, in contrast to pK15 CT^N2^ (Fig 1A). The inhibitory effect of the PLCγ1 cSH2 domain was more pronounced in the case of pK15 CT^N2^ (Fig 4C and 4D), suggesting that, in addition to the key contribution of the PLCγ1 cSH2 domain to the interaction with phosphorylated pK15, other regions in the pK15 CT and PLCγ1 SA domain may contribute.

We also investigated whether the isolated PLCγ1 cSH2 domain could interfere with the recruitment of PLCγ1 to pK15 in pK15-expressing cells. Co-transfection of full length pK15 together with a plasmid encoding the isolated PLCγ1 cSH2 domain resulted in a reduced phosphorylation of PLCγ1 on Y^783^, which is normally phosphorylated upon PLCγ1 activation (Fig 4E). In this experiment, the PLCγ1 cSH2 domain inhibited PLCγ1 phosphorylation to a greater extent than the PLCγ2 cSH2 domain (Fig 4E and 4F).

### Affinity of the distal pK15 SH2 binding site for PLCγ1 domains

**Fig 5:**
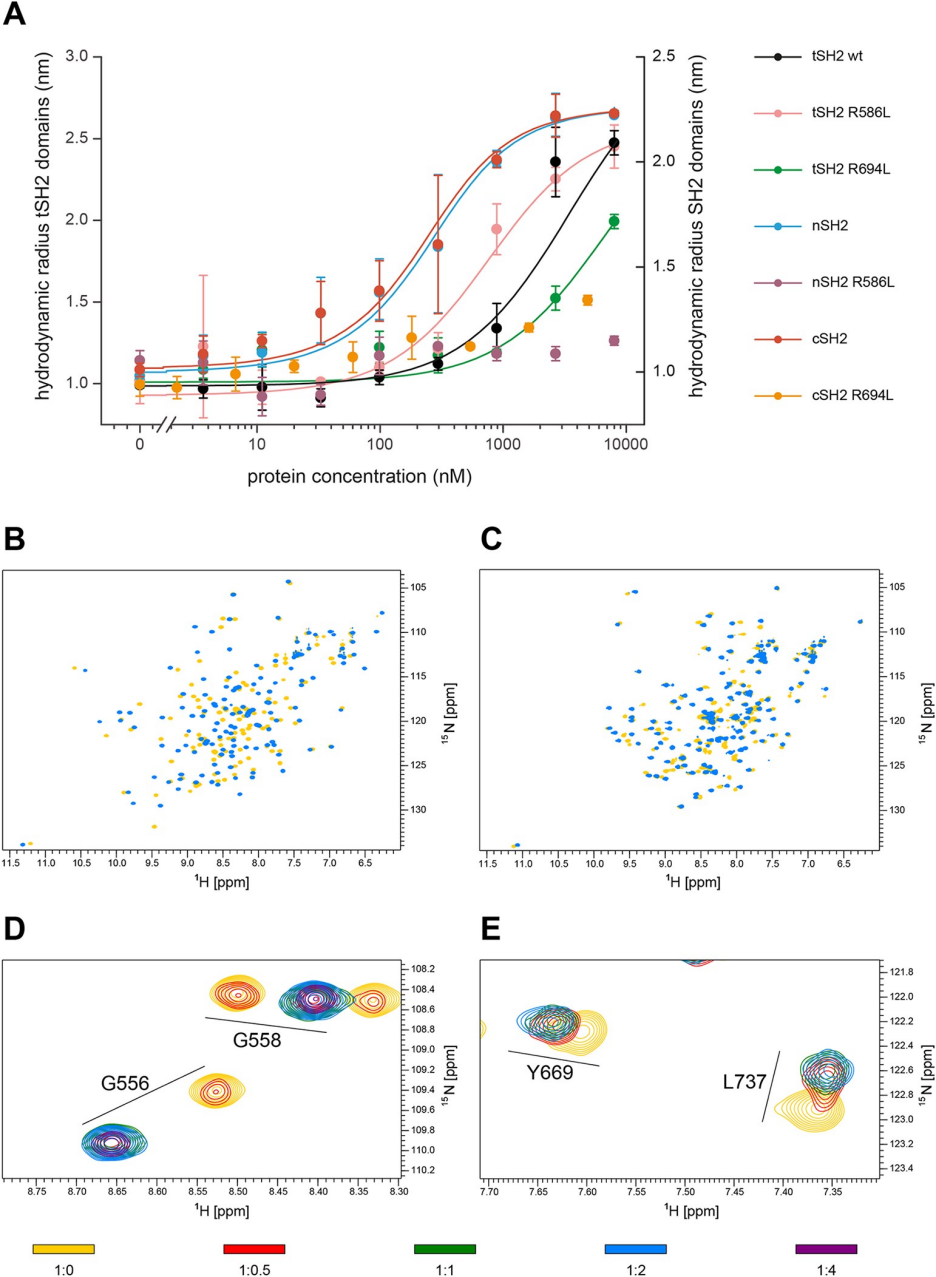
Affinity of pK15 peptide for PLCγ1 tSH2 or individual SH2 domains. (A) Binding curves of several PLCγ1 fragments in the presence of 200 nM 5,6-FAM-labelled pK15 (12mer). All measurements were performed in triplicate. The following affinities were measured: cSH2 (668–790): 166 ± 30 nM, nSH2 (545–662): 180 ± 43 nM, tSH2 (545–790): 1500 ± 450 nM, tSH2 R^694^L (545–790): 6318 ± 475 nM, tSH2 R^586^L (545–790): 440 ± 77. nSH2 R^586^L (545–662) and cSH2 R^694^L (668–790) do not bind to the peptide. (B and C) Overlay of ^1^H,^15^N HSQC NMR spectra of nSH2 (B) and cSH2 (C) alone (in yellow) and in the presence of 2 molar equivalents of the pK15 12mer peptide (blue), measured with a 600 MHz spectrometer at 298 K. The protein concentration was 100 μM and the buffer contained 100 mM MES, 150 mM NaCl, 3 mM TCEP, pH 6.5. (D and E) Excerpts from ^1^H-^15^N HSQC spectra of nSH2 (D) and cSH2 (E) upon addition of increasing concentrations of the pK15 12mer peptide. The peptide binds the nSH2 domain in the slow exchange regime, whereas it binds the cSH2 domain in the fast exchange regime.

We next investigated the affinity of a phosphorylated peptide representing the distal pK15 SH2 binding site and its key residues (Y^481^EEVL) for the PLCγ1 tSH2 domain. We used a phosphorylated FAM-labelled peptide representing the last 12 residues (DDLpYEEVLFPRN) of pK15 CT and measured its affinity to the purified PLCγ1 tSH2 domain (aa 545–790), to the R^586^L and R^694^L mutants of the tSH2 domain, and to the individual PLCγ1 SH2 domains (nSH2, aa 545–662; cSH2, aa 668–790) and their mutants (R^586^L and R^694^L), using a diffusion chamber approach. As shown in Fig 5A, the individual SH2 domains bound the phosphorylated pK15 peptide with high affinity (cSH2 domain, K_D_ = 166 ± 30 nM; nSH2 domain, K_D_ = 180 ± 43 nM), and mutation of the key arginine residues R^586^ and R^694^ in the isolated nSH2 and cSH2 domains, respectively, abolished pK15 peptide binding (Fig 5A), in keeping with the importance of the phosphorylated pK15 Y^481^ residue for the interaction with PLCγ1.

The PLCγ1 tSH2 domain bound the phosphorylated pK15 peptide ~10 times more weakly than the isolated nSH2 and cSH2 domains (K_D_ = 1500 ± 450 nM), suggesting that the tSH2 domain may be in a ‘closed’ conformation, which impedes access of the pK15 peptide to the individual SH2 domains. In spite of the similar affinities of the isolated nSH2 and cSH2 domains for the pK15 peptide, their contribution to the affinity of tSH2 was different. The tSH2 R^694^L mutant bound the pK15 peptide with a K_D_ of 6318 ± 475 nM, in agreement with the idea that the cSH2 subdomain is important for the interaction with the phosphorylated pK15 peptide, as also suggested by the results shown in Fig 4. However, the tSH2 R^586^L mutant, with a mutated pTyr recognition site in nSH2, bound the 12mer pK15 peptide better that WT tSH2 (K_D_ = 440 ± 77 nM) (Fig 5A). This result suggests that binding of the pK15 peptide to nSH2 in the context of the tSH2 fragment inhibits binding of pK15 to cSH2. The structural basis for this effect remains to be investigated.

Next, we used Nuclear Magnetic Resonance (NMR) spectroscopy to further compare the interaction of the phosphorylated pK15 12mer to the isolated nSH2 and cSH2 domains (Fig 5B–5E). We recorded ^1^H-^15^N HSQC spectra of each of the two domains in the presence of increasing concentrations of the pK15 peptide. In this spectrum, each N-H group of the protein gives rise to one peak. Thus, besides the side-chain amide groups, the spectrum shows one peak per amino acid. A change in the position of a few NMR peaks of the amide groups of the protein upon addition of the peptide is indicative of binding. We could confirm that both domains bound the K15 peptide with similar affinity, as the NMR peaks of either nSH2 or cSH2 had completely shifted to the frequencies of the corresponding protein–peptide complex after addition of similar amounts of peptide (that is, the saturating concentration of the peptide was the same). The behavior of the NMR peaks during the titration series, however, differed for the two SH2 domains, indicating different binding kinetics. In NMR the way how peaks change their position during a titration experiment depends on the exchange constant k_ex_, defined as the sum of the dissociation rate k_off_ and the association rate k_on_ of the complex (k_ex_ = k_off_ + k_on_). If k_ex_ is more than one order-of-magnitude larger than the difference in the frequencies of the peptide-free and peptide-bound states of the protein (that is, the difference in the position of the peak in the absence of peptide and in the presence of a saturating concentration of peptide), the peak is expected to move continuously from the peptide-free to the peptide-bound position during the titration (fast exchange regime), as seen in Fig 5E. However, if k_ex_ is more than one order-of-magnitude smaller than the difference in the frequencies of the peptide-free and peptide-bound states of the protein, the peak is expected to disappear from the peptide-free position and reappear at the peptide-bound position during the titration (slow exchange regime). At a saturating concentration of peptide, the peak has completely disappeared from the peptide-free position and has gained maximum intensity at the peptide-bound position, as in Fig 5D. Thus, the spectra in Fig 5D and 5E demonstrated that either the dissociation rate k_off_ or association rate k_on_ of the nSH2–peptide complex is more than one order of magnitude slower than the corresponding rate of the cSH2–peptide complex. In light of this, it is conceivable that the preference of pK15 CT for cSH2 *in vivo* may be determined by kinetic rather than thermodynamic factors.

### Spatial organization of the PLCγ1 tSH2 domain in complex with the distal pK15 SH2 binding site

**Fig 6:**
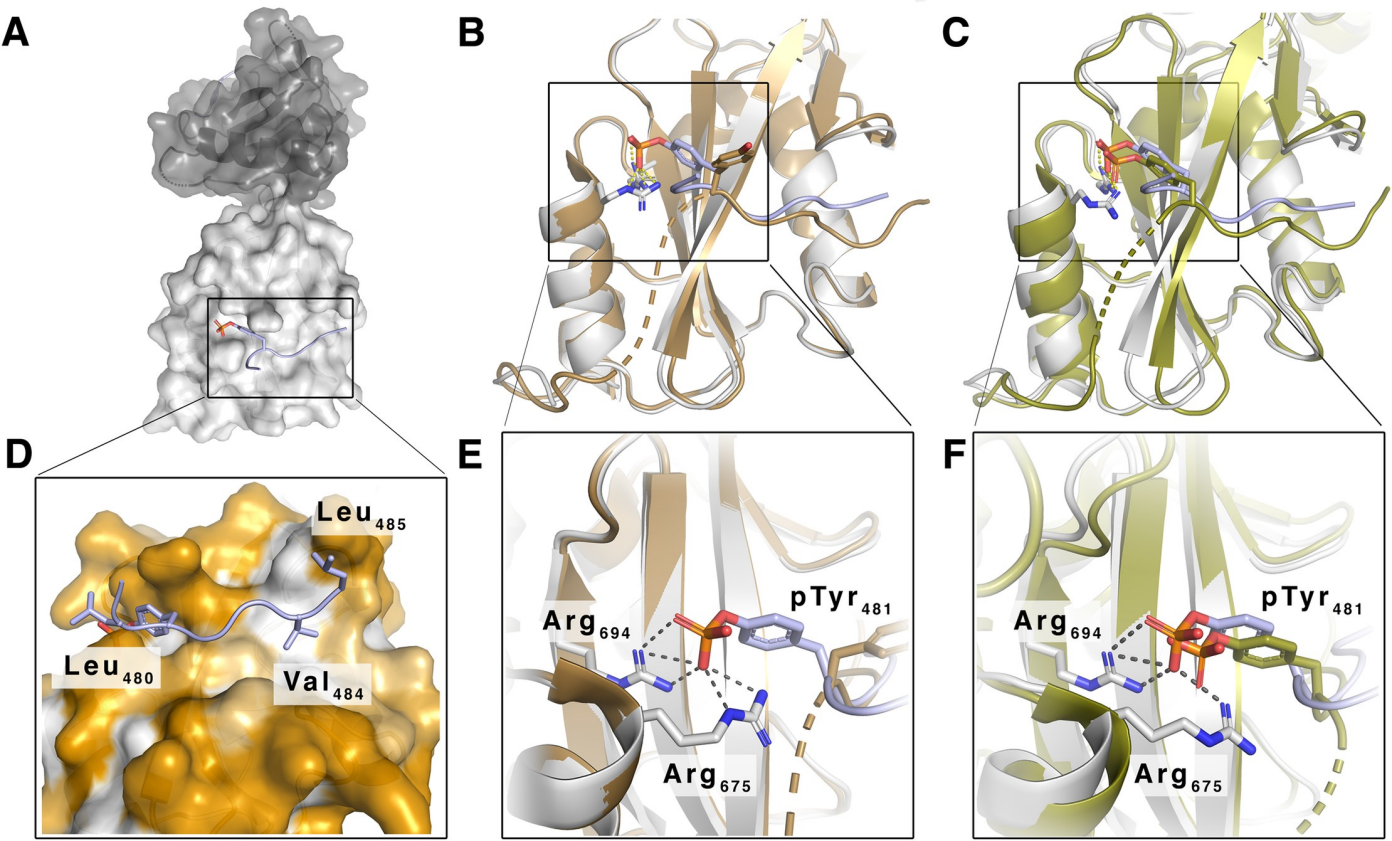
Structure of the last 12 amino acids of pK15 CT in complex with the PLCγ1 tSH2 domain. (A) Surface representation of the truncated PLCγ1 tSH2 domain with the nSH2 domain shown in dark grey and the cSH2 domain shown in light gray. The pK15 12mer peptide bound to the cSH2 domain is shown as cartoon in light blue with the side chain of Y^481^ shown as sticks and the phosphorylation colored in red and orange for oxygen and phosphorus atoms, respectively. (B+C) Cartoon representation of the cSH2 domain (shown in gray) in complex with the pK15 12mer peptide (shown in light blue) superimposed on (B) the published structure of the tSH2 domain including the unphosphorylated linker segment (shown in brown, PDB 4FBN) between the cSH2 domain and the SH3 domain and (C) the analogous structure with a phosphorylated linker segment (shown in olive, PDB 4EY0) [46,52]. The amino acid backbone of the pK15 12mer peptide superposes surprisingly well on the backbone of the linker peptide between cSH2 and SH3 domains. The unphosphorylated Y^783^ sidechain in the cSH2-SH3 linker segment is oriented away from the phosphotyrosine-binding pocket of the cSH2 domain, while the phosphorylated Y^783^ sidechain superposes well with the K15Y^481^ side chain. (D) Surface representation of the binding pocket of the PLCγ1 cSH2 domain showing the interaction of hydrophobic residues L^480^, V^484^, L^485^ in the phosphorylated pK15 12mer with the cSH2 domain. Hydrophobic residues are colored in white, hydrophilic residues in orange. (E+F) Close-up of the phosphotyrosine-binding pocket of the cSH2 domain shown in B+C. The docked phosphorylated Y^481^ sidechain (light blue/red) of the pK15 12mer peptide, which makes multiple polar contacts with the sidechains of R^675^ and R^694^ (shown as sticks with carbon atoms colored in gray and nitrogen atoms in blue) at its bottom.

To obtain structural insights into this interaction we sought to determine the structure of the PLCγ1 tSH2 domain in complex with the phosphorylated 12mer pK15 peptide. Initial attempts to obtain diffractable crystals of the entire PLCγ1 tSH2 domain (aa 541–790) [46,52] in complex with this 12mer pK15 peptide were unsuccessful. We reasoned that the linker region separating the PLCγ1 cSH2 and SH3 domains (Fig 1B) could have interfered with the binding of the pK15 12mer peptide to the PLCγ1 cSH2 domain, since the former contains tyrosine residue 783, which is known to interact with the PLCγ1 cSH2 domain [46]. We therefore expressed and purified a truncated PLCγ1 tSH2 domain (PLCγ1 aa 545–772), which lacks this linker segment. We obtained diffraction quality crystals of this truncated tSH2 domain in complex with the 12mer phosphorylated pK15 peptide. The structure was determined by the molecular replacement method and refined to a resolution of 2.1Å (Fig 6). Details of crystallization and structure determination are described in Materials and Methods, and the crystallographic statistics are listed in Table 1. In this complex, the 12mer pK15 peptide is unambiguously bound to the PLCγ1 cSH2 domain (Fig 6A and 6B), but at lower occupancy peptide binding to the nSH2 domain was also observed, in line with the findings shown in Figs 3–5. In the PLCγ1 cSH2 domain the pK15 peptide straddles the triple ß-sheet that divides the two pockets of the SH2 domain (Fig 6B). Its phosphorylated tyrosine residue (pK15 Y^481^) is in contact with PLCγ1 R^675^ and R^694^ in the first of these two pockets (Fig 6E and 6F), while its hydrophobic residues V^484^ and L^485^ in the canonical pK15 SH2-binding site (YEEVL) are in proximity to the second pocket that is known to accommodate hydrophobic residues (Fig 6D). The pK15 peptide adopts a similar position to that of residues 781–790 in the PLCγ1 linker peptide containing phosphorylated Y^783^, which connects the PLCγ1 tSH2 and SH3 domains (Figs 1B, 6C, and 6F) [46]. In its unphosphorylated form, Y^783^ in the PLCγ1 tSH2 domain linker is oriented in the opposite direction in the published structure of the PLCγ1 tSH2 domain [52] (Fig 6B and 6E). We therefore propose that, following phosphorylation of pK15 on Y^481^, the last 12 amino acids of the pK15 cytoplasmic domain engage the PLCγ1 cSH2 domain in a manner similar to the PLCγ1 tSH2-SH3 domain linker, once the latter has been phosphorylated on Y^783^ by the activating receptor tyrosine kinase (Figs 1B, 6B, 6E and 6F).

**Table 1:** Data collection, phasing and refinement statistics.

	PLCg tSH2 / K15 peptide
**Data collection**	
Space group	*P* 2_1_ 2_1_2
Cell dimensions	
*a*, *b*, *c* (Å)	54.66 77.03 59.96
*a*, *b*, *g* (°)	90.0 90.0 90.0
Molecules in AU	1
Resolution (Å)	50–2.1 (2.22–2.1)^a^
*R*_meas_	0.085 (0.809)
*I /* σ (*I*)	12.19 (1.83)
*CC*_1/2_	0.998 (0.895)
Completeness (%)	99.5 (97.8)
Redundancy	7.07 (7.25)
**Refinement**	
Resolution (Å)	44.58–2.08 (2.175–2.1)
No. reflections	15299
*R*_work_ / *R*_free_	0.2216 / 0.2788
No. atoms	
Protein	1897
Solvent	32
*B* factors	
Protein	62.52
Solvent	51.51
R.m.s deviations	
Bond lengths (Å)	0.013
Bond angles (°)	1.57
Ramachandran plot^§^	
Favoured (%)	98.12
Allowed (%)	1.88
Outliers (%)	0
Rotamer outliers (%)	1.98

A single crystal was used to collect the diffraction data set used to determine the structures.

a Values in parentheses are for highest-resolution shell.

§ Ramachandran statistics were calculated with MolProbity

### Binding of phosphorylated pK15 CT enhances phosphorylation of PLCγ1 by src kinase

During its recruitment to the phosphorylated C-terminal kinase domains of receptor tyrosine kinases (RTK), PLCγ1 is thought to bind to the RTK via its nSH2 domain, thereby allowing the phosphorylation of PLCγ1 Y^783^, which then in turn engages the cSH2 domain to trigger a conformational change in PLCγ1 that is required for its lipase activity [43,46,52]. However, an alternative model envisages engagement of both the nSH2 and cSH2 domains by the activated RTK [47]. Our observation that phosphorylated pK15 CT, which does not possess kinase activity, engages the PLCγ1 cSH2 domain therefore raised the question how this would impact on the phosphorylation of PLCγ1. The scaffold protein SLP-76 uses its non-canonical SH2 binding site (pY^173^IDR) to interact with the PLCγ1 cSH2 domain [53] and a proline-rich motif (PPVPPQRP) to bind to the PLCγ1 SH3 domain [54]. The interaction between SLP-76 pY^173^IDR and the PLCγ1 cSH2 domain has been hypothesized to promote a conformational change in PLCγ1 that displaces the autoinhibitory cSH2 domain from the PLCγ1 core and thereby leads to an increased exposure and enhanced phosphorylation of PLCγ1 on Y^783^ in the cSH2-SH3 linker peptide (Fig 1B) by the TEC family kinase ITK [53]. We therefore asked if pK15 could recruit and activate PLCγ1 in a manner similar to SLP-76.

**Fig 7:**
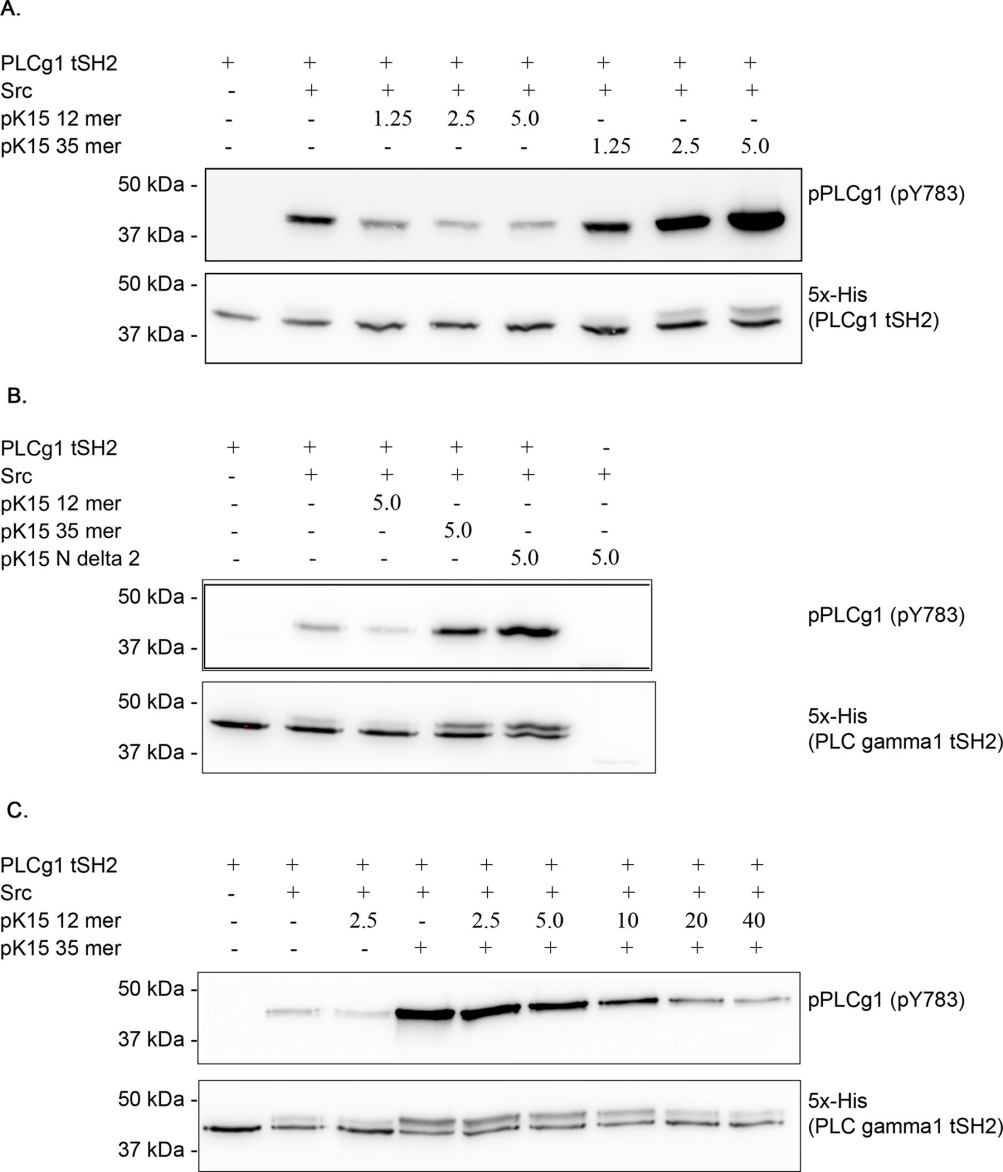
The pK15 CT domain enhances Src-mediated phosphorylation of the PLCγ1 tSH2 domain. (A) The purified PLCγ1 tSH2 domain (0.6 μM) was incubated with limiting amounts (0.085 μM) of recombinant purified Src kinase expressed in insect cells in the presence of increasing concentrations (1.25–5 μM) of phosphorylated peptides representing the last 12 (pK15 12mer) or 35 (pK15 35mer) amino acids of the pK15 cytoplasmic domain for 1 hour at 37°C. The reaction was stopped using SDS sample buffer and analysed by SDS-PAGE and Western blot using an antibody to the PLCγ1 pY^783^ residue (top panel) or to the His tag on the recombinant tSH2 domain (bottom panel). (B) Same as above, except that a fixed concentration (5.0 μM) of the 12mer peptide, the 35mer peptide or the pK15 truncation construct GST-pK15 CT^N2^ [51] were used. The increased phosphorylation of the tSH2 domain in the presence of the 35mer peptide and pK15 CT^N2^ can be seen. (C) Increasing concentrations of the phosphorylated pK15 12mer peptide were used to inhibit the increased src-mediated phosphorylation of tSH2 induced by 2.5 μM of the pK15 35mer peptide. Experimental conditions were as in panel A.

To address this hypothesis, we tested if phosphorylated fragments of pK15 CT could enhance the phosphorylation of the purified PLCγ1 tSH2 domain on Y^783^ by recombinant Src kinase in an *in vitro* kinase assay. We established conditions in which limiting amounts of recombinant purified Src kinase were added to the purified tSH2 domain so as to allow only a low level of tSH2 domain phosphorylation, which we detected on Western blots using an antibody to the phosphorylated PLCγ1 Y^783^ motif. Using these conditions, we then tested whether increasing amounts of the phosphorylated 12mer pK15 peptide used in the crystallography, diffusion chamber and NMR experiments (Figs 5 and 6), a longer phosphorylated pK15 peptide corresponding to the last 35 amino acids of pK15 CT, or the in vitro phosphorylated pK15 CT^N2^ could enhance the Src-mediated phosphorylation of the PLCγ1 tSH2 domain. While the 12mer pK15 peptide showed a moderate inhibition in this assay (Fig 7A and 7B), the 35mer pK15 peptide enhanced the Src-mediated phosphorylation of the PLCγ1 tSH2 domain in a dose dependent manner, as shown by the increased intensity of the tSH2 band stained with an antibody to the phosphorylated PLCγ1 Y^783^ motif (Fig 7A, upper panel) and the appearance of a more slowly migrating tSH2 band on the Western blot stained with an antibody to the 5x His tag on the recombinant tSH2 protein (Fig 7A, lower panel). Likewise, the in vitro phosphorylated pK15 CT^N2^ protein, which corresponds to the last 80 amino acids of pK15 CT (see above), also enhanced the Src-mediated phosphorylation of the PLCγ1 tSH2 domain (Fig 7B). This result suggests that, like SLP-76, phosphorylated pK15 might act as a scaffold protein that recruits PLCγ1 via its cSH2 domain and thereby promotes its activation by a tyrosine kinase.

### Inhibition of the pK15 CT-mediated enhancement of PLCγ1 phosphorylation using a short pK15-derived peptide

Unlike the C-terminal 80 or 35 amino acids of the pK15 CT domain, the shorter 12mer peptide cannot enhance the Src-mediated phosphorylation of PLCγ1 (Fig 7A and 7B). We therefore explored if this short 12mer peptide, which contains the binding site for the PLCγ1 cSH2 domain (Fig 6) can inhibit the pK15-mediated enhancement of PLCγ1 phosphorylation in a competitive manner. As shown in Fig 7C, the phosphorylated 12mer peptide could inhibit the increased PLCγ1 tSH2 phosphorylation induced by the 35mer peptide. This observation suggests that it may be possible to counteract the pK15-dependent phosphorylation and activation of PLCγ1 with small peptides and possibly also with small pharmacological inhibitors.

## Discussion

The role of KSHV as causative agent of Kaposi’s sarcoma was established more than twenty years ago [1] and since then substantial evidence has accumulated demonstrating the contribution of different viral proteins and cellular pathways to the disease progression. The ability of KSHV to trigger invasiveness and angiogenesis is important for the development of KS and is associated with higher expression of matrix metalloproteinases (MMPs), activation of cyclooxygenase 2 (Cox-2), mitogen-activated protein kinase kinase kinase kinase 4 (MAP4K4), PI3K/Akt/mTOR, NF-κB and PLCγ1 pathways [29,38,55–59]. Also viral factors increasing angiogenesis and invasion have been described: viral microRNAs miR-K6-3p and miR-K6-5p as well as proteins pK1, LANA, vFLIP, vGPCR and pK15 among others [29,38,59–63]. We have previously shown the importance of the recruitment and activation of PLCγ1 by pK15 during the reactivation of KSHV from latency [28] and the increased migration, proliferation and invasiveness of KSHV-infected endothelial cells [28,29,38]. A better understanding of how pK15 recruits and activates PLCγ1 might therefore provide a basis for the development of small molecule inhibitors that target some of these effects induced by KSHV in endothelial cells.

In this study we first demonstrate that pK15 is phosphorylated on tyrosine residues in KSHV-infected cells (Fig 1). While such tyrosine phosphorylation of pK15 has previously been shown with the help of an overexpressed chimeric K15 protein [24] and the purified recombinant pK15 CT [31], the result shown in Fig 1 demonstrates that phosphorylation of pK15 on tyrosine residues occurs in KSHV-infected epithelial and endothelial cells. We also show that pK15 phosphorylation on Y^481^ in the second SH2 binding site (Y^481^EEV) of the pK15 CT enhances binding of pK15 to PLCγ1 (Fig 2). This observation is in line with the fact that pK15 can be phosphorylated on Y^481^ by Src family kinases *in vitro* and that the SH2 binding site is necessary for PLCγ1 recruitment [25,31,37,38].

PLCγ1 contains two SH2 domains, which contribute differentially to its interaction with other proteins. Both its nSH2 and cSH2 domains are necessary for the interaction of PLCγ1 with platelet-derived growth factor receptor (PDGFR) [64], while the nSH2 domain mediates the interaction with the phosphorylated cytoplasmic domains of the epidermal growth factor receptor (EGFR) and fibroblast growth factor receptor (FGFR) [46,52,65]. The cSH2 domain has been reported to also bind directly to the FGFR cytoplasmic domain [47] and to an atypical SH2-binding site motif (pY^173^IDR) of SLP-76 [53]. The prevailing view holds that the cSH2 domain is required for an intramolecular interaction with the phosphorylated Y^783^ residue in the linker domain between the cSH2 and SH3 domains of PLCγ1, which relocates the cSH2 domain from an inhibitory interaction with the PLCγ1 core and is thus thought to contribute to conformational changes in PLCγ1 that are required for its enzymatic activity [43,46,66].

In our experiments with the purified phosphorylated pK15 CT domain, PLCγ1 cSH2 seemed to be the main contributor to its interaction with pK15 (Figs 4A, 4B, 5 and 6). When we used the purified, in vitro phosphorylated, GST-fused pK15 CT domain to pull down full length PLCγ1 expressed in mammalian cells, mutations of arginine residues in the phosphotyrosine-binding pocket of individual PLCγ1 SH2 domains reduced their interaction (Fig 3). However, only in the case of the PLCγ1 SA domain did the simultaneous mutation of both the nSH2 and the cSH2 domain completely abolish the interaction with phosphorylated pK15 (Fig 3B), suggesting that PLCγ1 regions outside the SA domain may contribute to the recruitment of PLCγ1 to phosphorylated pK15. Our results obtained with transfected cells are therefore compatible with both the nSH2 and the cSH2 domain contributing to the pK15—PLCγ1 interaction. However, when isolated PLCγ1 domains were expressed in cells, or used as purified recombinant proteins, and tested for the interaction with phosphorylated pK15, the PLCγ1 cSH2 domain bound to pK15 CT more strongly than the nSH2 domain, both in cells and *in vitro* (Figs 3C, 4A and 4B). Also, the cSH2 domain in isolation inhibited the interaction of pK15 with PLCγ1 (Fig 4C and 4D) as well as pK15-induced PLCγ1 phosphorylation in transfected cells (Fig 4E).

We measured the affinity of a short phosphorylated pK15 peptide, representing the last 12 amino acids of pK15 CT, for the entire PLCγ1 tSH2 domain, as well as for its individual SH2 domains. Our results suggest that the entire PLCγ1 tSH2 domain has a low affinity for this short peptide, while individual SH2 domains display a higher affinity (Fig 5). It therefore appears that the entire PLCγ1 tSH2 domain adopts a conformation that is only poorly accessible to pK15 CT. This hypothesis is supported by our observation that we had to truncate the tSH2 domain linker containing PLCγ1 Y^783^ in order to obtain diffraction quality crystals of the tSH2 domain in complex with this 12mer pK15 peptide (Fig 6). A comparison of the previously published structure of the entire PLCγ1 tSH2 domain [46,52] with the structure of the truncated PLCγ1 tSH2 domain in complex with the 12mer pK15 peptide indicates that the C-terminal end of the pK15 cytoplasmic domain may compete with the PLCγ1 cSH2-SH3 linker for binding to the PLCγ1 tSH2 domain (Fig 6B). If we assume that the cSH2-SH3 linker is able to bind to the cSH2 domain better in the tSH2 fragment than in the isolated cSH2 domain, this interpretation would explain the higher affinity of the isolated cSH2 domain compared to the entire tSH2 domain for the phosphorylated last 12 amino acids of pK15 CT (Fig 5A).

While the pK15 12mer peptide showed comparable affinities to the isolated PLCγ1 nSH2 and cSH2 domains (Fig 5A), longer pK15 CT fragments bound much more strongly to the cSH2 domain than to the nSH2 domain (Fig 4A and 4B). This could suggest that other regions in the pK15 CT domain may also contact the PLCγ1 cSH2 and thereby increase the affinity of the phosphorylated pK15 CT domain for the PLCγ1 cSH2 relative to the nSH2 domain. In addition, by NMR we found that the 12mer peptide showed a faster exchange with the cSH2 domain than with the nSH2 domain (Fig 5B–5E), providing the possibility of a kinetically-driven binding regulation *in vivo*.

**Fig 8:**
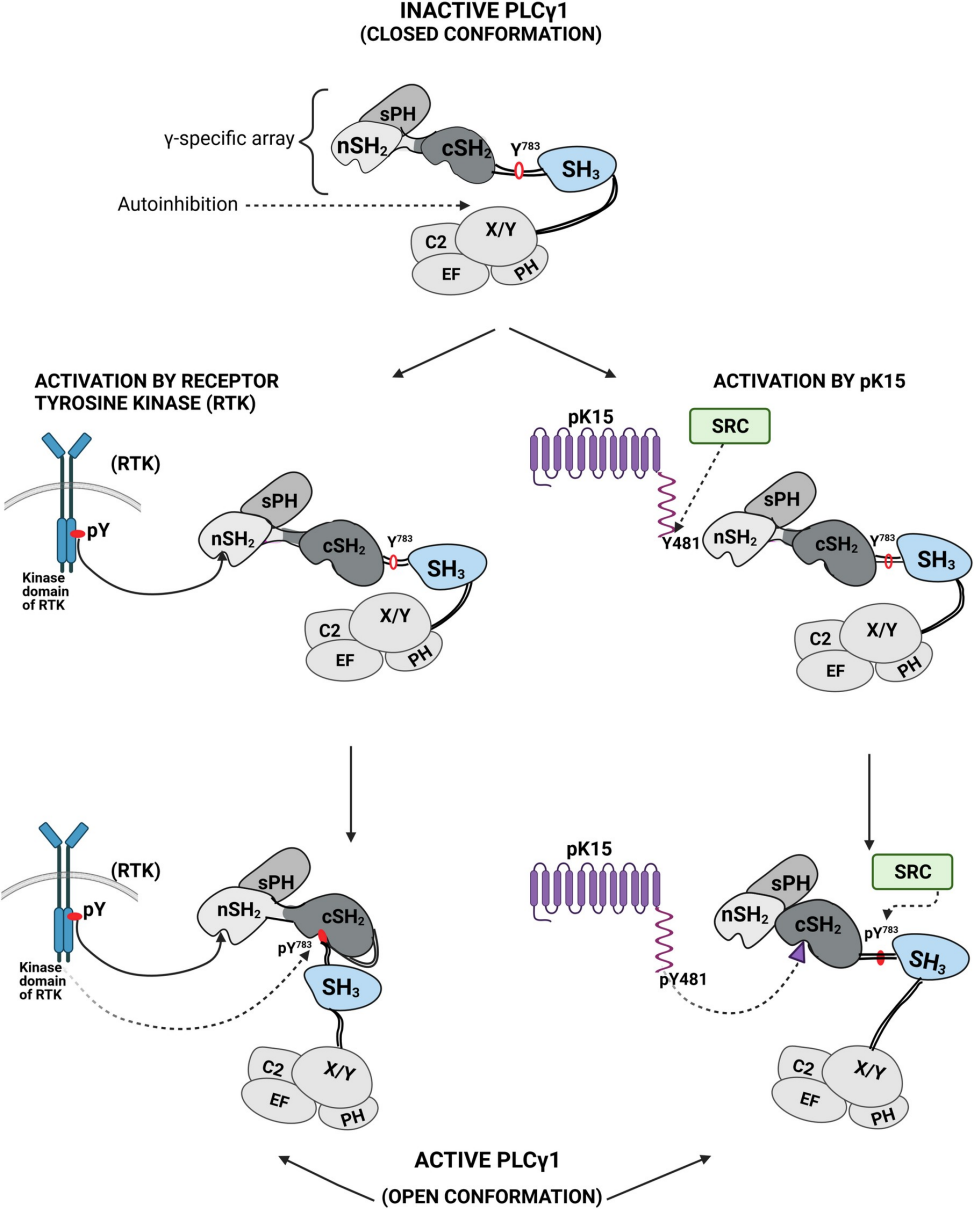
Diagram illustrating the activation of PLCγ1 by receptor tyrosine kinases or pK15. Top: Model of PLCγ1 showing individual PLCγ1 domains. nSH2, cSH2: N-terminal and C-terminal SH2 domain; SH3: SH3 domain; sPH: split PH domain; C2: C2 domain; EF: EF domain; X/Y: catalytic core domain. PLCγ1 is depicted in its closed, enzymatically inactive conformation, in which the cSH2 domain is positioned in the proximity of the catalytic core (X/Y). The model is based on findings reported in references [43,44,45,46,66]. Below Left: Activation of PLCγ1 by receptor tyrosine kinases (RTK): following autophosphorylation on tyrosine residues, the cytoplasmic RTK domain recruits the PLCγ1 nSH2 domain and phosphorylates PLCγ1 Y^783^, which in turn binds into the cSH2 domain, thus removing it from its proximity to the catalytic core, and thereby activates the enzymatic activity of the latter. Below Right: Activation of PLCγ1 by pK15: as shown in this report, the phosphorylated Y^481^ of pK15 (phosphorylated by Src in our experiments) binds into the PLCγ1 cSH2 domain. This presumably leads to a conformational change in PLCγ1, which in turn facilitates the subsequent phosphorylation of PLCγ1 Y^783^ by members of the Src kinase family and subsequent activation of PLCγ1 lipase activity.

We also found that the last 35 amino acids of the cytoplasmic domain of pK15, as well as a longer 80 amino acid segment derived from the C-terminal end of the pK15 cytoplasmic domain, can enhance the phosphorylation of PLCγ1 by Src kinase in vitro (Fig 7). A possible interpretation of this observation is that the pK15 cytoplasmic domain displaces the PLCγ1 cSH2-SH3 linker peptide (Figs 1B and 6B) from the PLCγ1 cSH2 domain and thus renders Y^783^ in this PLCγ1 linker peptide more accessible to Src-mediated phosphorylation, as schematically depicted in Fig 8. The fact that the short 12mer pK15 peptide does not increase the Src-mediated phosphorylation of the PLCγ1 tSH2 domain suggests that pK15 CT regions outside the 12mer peptide may contribute to this conformational change. We find it intriguing that a similar mechanism of PLCγ1 activation has been reported for the SLP-76 scaffold protein, which, like pK15, uses its SH2-binding site (pY^173^IDR) to bind to the PLCγ1 cSH2 domain and thereby primes PLCγ1 for phosphorylation by the T-cell receptor associated tyrosine kinase ITK [53]. We therefore speculate that pK15 mimics aspects of intracellular signaling triggered by the activation of the T cell receptor. Interestingly, the positional homologue of the K15 gene in the related gammaherpesvirus Epstein-Barr virus (EBV) is LMP2, and the LMP2A protein is thought to mimic aspects of B-cell receptor signaling [67]. The two non-structural membrane proteins pK15 and LMP2A of these two human gammaherpesviruses may thus have found similar ways to engage intracellular pathways involving the activation of PLCγ1. Intriguingly, the cagA protein of *Helicobacter pylori* is also phosphorylated by Src family kinases, allowing phosphorylated cagA to recruit and activate the cellular phosphatase SHP2 [68]. Since we could show that, in the case of KSHV pK15, the increased phosphorylation of PLCγ1 observed in the presence of a pK15 fragment can be inhibited with a small peptide that docks into the PLCγ1 cSH2 domain (Figs 6 and 7), the underlying mechanism may provide a target for the future development of inhibitors against KSHV-related diseases. The structure of this short pK15 peptide in complex with the PLCγ1 tSH2 domain reported here provides the basis for the *in silico* screening of small molecule inhibitors that would interfere with the pK15—PLCγ1 interaction by docking into the PLCγ1 cSH2 pocket shown in Fig 6. Given the key role of the phosphorylated PLCγ1 Y^783^ residue in the PLCγ1 cSH2-SH3 linker peptide during the activation of PLCγ1 by cellular receptor tyrosine kinases (Fig 8), we speculate that small molecules mimicking the effect of the pK15 12mer peptide described here could also prove useful in controlling PLCγ1 activity in other diseases that are caused by an excessive activation of PLCγ1 [43,69].

## Materials and methods

### DNA constructs

pFJ-EA and pFJ K15P WT were a kind gift from J. Jung (University of Southern California). The generation of the pFJ K15P YF mutant was described before [31]. pGEX-6P-1 K15P 347–489 is described in [29]. N-terminal truncation mutants of K15 cytoplasmic tail pGEX 6P-1 K15P N1 and N2 were generated by PCR on pGEX-6P-1 K15P 347–489, using forward primers 5’-GCATTGGATCCATTTATACCCGTGATCAGAATCTGC-3’ and 5’-GCATTGGATCCAGCCAGCCGCTGAATGAAG-3’ respectively. In both cases the reverse primer 5’-GCATTGAATTCTTAGTTACGCGGAAACAGAACTTCT-3’ was used. The resulting products were inserted in pGEX 6P-1 using BamHI/EcoRI. pGEX 4T-1 ex8 K15P YF was described in [25].

PLCγ1 full length (FL), specific array (SA) in pTriEx-4 vector, tandem SH2 (tSH2) in pOPINS vector and PLCγ2 cSH2 in pTriEx-4 were kindly provided by M. Katan (University College London). PLCγ1 FL with mutated arginines (R586L, R694L or R586L+R694L, double mutant, DM) were described before [29]. PLCγ1 SA R586L was generated with primers 5’-CTTCCTCGTGCTAGAGAGTGAG-3’ and 5’-CTCACTCTCTAGCACGAGGAAG-3’. PLCγ1 SA R694L was generated with primers 5’-CCTTCCTGGTGCTGAAGCGGAATGAACCC-3’ and 5’-GGGTTCCTTCCGCTTCAGCACCAGGAAGG-3’. PLCγ1 SA DM was generated on PLCγ1 SA R586L with primers for PLCγ1 SA R694L. PLCγ1 nSH2 was generated by PCR from PLCγ1 FL with primers 5’-CGCGGATCCACACTCCAATGAGAAGTGGTTCC-3’ and 5’-GCGAATTCAATTACTCTTTGCTCTCGTGGGCG-3’. PLCγ1 cSH2 was generated by PCR from PLCγ1 FL with primers 5’-CGGGATCCAAACGCCCACGAGAGCAAAGAG-3’ and 5’-GCGAATTCAATTACAGGGCCCCGTAGTCAGGCTC-3’. For the generation of PLCγ1 nSH2 R586L, PLCγ1 cSH2 R694L, PLCγ1 tSH2 R586L, PLCγ1 tSH2 R694L and PLCγ1 tSH2 DM, the same primers were used as for the corresponding mutants in PLCγ1 SA. PLCγ1 cSH2 for the AlphaLISA experiment was cloned from pTriEx-4 PLCγ1 cSH2 with primers 5’-CGGGATCCAACGCCCACGAGAGCAAAGAG-3’ and 5’-CGCTCGAGTTACAGGGCCCCGTAGTCAG-3’ into the Strep tag-containing pET31 vector using BamHI/XhoI.

A synthetic gene (Eurofins Genomics, Germany) encoding the SA was used as a starting point for cloning the constructs for the affinity measurements (PLCγ1 nSH2, PLCγ1 cSH2, PLCγ1 tSH2, PLCγ1 nSH2 R586L, PLCγ1 cSH2 R694L, PLCγ1 tSH2 R586L and PLCγ1 tSH2 R694L). The constructs were cloned in pETM22 using a restriction enzyme-free approach [70]. The mega-primer for PLCγ1 nSH2 was generated with primers 5’-GAAGTTCTGTTCCAGGGGCCCCATAGCAATGGTTTCATGGCAAACTTGG-3’ and 5’-GCAAGCTTGTCGACGGAGCTCTTAATTCCTCTGCGGCACCGG-3’. The mega-primer for PLCγ1 cSH2 was generated with primers 5’-GAAGTTCTGTTCCAGGGGCCCCATGAAAGCAAAGAGTGGTATCACGC-3’ and 5’-GCAAGCTTGTCGACGGAGCTCTTACGGCATCGGATTTGCTTCAAC-3’. The mega-primer for PLCγ1 tSH2 was generated with primers 5’-GAAGTTCTGTTCCAGGGGCCCCATAGCAATGGTTTCATGGCAAACTTGG-3’ and 5’-GCAAGCTTGTCGACGGAGCTCTTACGGCATCGGATTTGCTTCAAC-3’. The mega-primers generated from the first PCR were used for a second restriction enzyme-free PCR that allows the insertion into the destination plasmid (pETM22). Then, the parental plasmid was digested with DpnI (NEB). Mutations were generated using PCR-mediated mutagenesis (Agilent, QuickChange site-directed mutagenesis manual).

Full length chicken Src was cloned from pcDNA3.1 Myc-His (-) Src (provided by S. Lang) using primers 5’-CGCGGATCCGATCTCAATATGGGGAGCAGCAAGAGCAAG-3’ and 5’-CGCTCTAGATAGGTTCTCTCCAGGCTGG-3’ and inserted into the pT1204 vector.

### Protein purification

For production and purification of GST-fused K15 proteins, Rosetta E. coli were grown overnight in LB-medium with ampicillin at 37°C, 220 rpm, then diluted 1:10 in fresh LB containing ampicillin and incubated until OD_600_ 0.6. Protein production was induced with 1 mM IPTG for 4 hours at 30°C. Cells were pelleted at 5.000g 4°C for 10 minutes, resuspended in PBS (50ml for 1 liter culture) with protease inhibitors and lysed by sonication on ice 5 times for 30 seconds with a 30 second pause after each sonication and 0.5% (v/v) NP-40 was then added. After centrifugation at 30.000g, 4°C for 10 minutes, the supernatant was transferred to a new tube for the second centrifugation. Cleared lysate was incubated overnight at 4°C on a roller with Glutathione Sepharose 4 Fast Flow beads (GE Healthcare), which were washed before 3 times in PBS. Afterwards, beads with bound protein were washed 3 times in 1xPBS, 5% (v/v) glycerol, 0.5% (v/v) NP-40, protease inhibitors and the protein was eluted by incubating the beads for 3 hours at 4°C on a roller in 1xPBS, 10% (v/v) glycerol, 0.5% (v/v) NP-40, 60 mM glutathione, protease inhibitors, pH7.3. The eluted material was centrifuged for 10 minutes at 3.220g and dialyzed against PBS with 1 mM DTT and 100 μM PMSF in a Slide-A-Lyzer Dialysis Cassettes, 3.5K MWCO (Thermo Fisher Scientific).

For expression and purification of PLCγ1 constructs cloned as a thioredoxin fusion protein in pETM22, the plasmids were transformed into *E*. *coli* strain BL21(DE3). Cultures were grown in Luria-Bertani (LB) broth at 37°C to OD_600_ of 0.6, then induced with 1 mM IPTG and incubated at 20°C overnight. ^15^N-labeled proteins for NMR studies were prepared by growing the bacteria in M9 minimal medium containing kanamycin (50 μg/ml) and ^15^NH_4_Cl (1 g/l, Cambridge Isotope Laboratories). Cells were harvested and resuspended in lysis buffer (1 M NaCl, 50 mM Tris-HCl, 5% glycerol, 10 mM imidazole, 5 M mercaptoethanol, 1 EDTA-free protease inhibitor cocktail tablet (Roche), 100 μg lysozyme (Roth), 50 μg DNAse (NEB), pH 7.6) then lysed by sonication, followed by centrifugation to remove cellular debris. The filtered supernatant was loaded on a HisTrap HP column (GE Healthcare) pre-equilibrated with wash buffer. The thioredoxin tag was cleaved with 3C protease overnight and the cleaved protein was collected in the flow-through of a second Ni^2+^-affinity chromatography run. The eluted sample was subsequently subjected to size-exclusion chromatography on HiLoad 16/600 Superdex 75pg column (GE Healthcare) pre-equilibrated with gel filtration buffer (100 mM MES, 150 mM NaCl, 3 mM TCEP (Tris(2-carboxyethyl)phosphin, Roth), pH 6.5).

PLCγ1 constructs with 6xHis in pTriex-4 and pOPINS vectors were grown as described for GST constructs, but kanamycin was used instead of ampicillin for pOPINS. Bacteria were grown until OD_600_ 0.8 and the temperature was changed to 30°C. When the OD_600_ reached 1, protein production was induced with 1 mM IPTG for 5 hours at 30°C, bacteria were pelleted at 6.000g, 4°C for 10 minutes and the pellet was stored at -80°C. After pellet resuspension in 40 ml buffer core buffer (50 mM Tris pH 7.4, 500 mM NaCl, 0.1% (v/v) Triton X-100, protease inhibitors), cells were lysed by sonication on ice 5 times for 30 seconds with 30 seconds pause after each sonication and the lysate was cleared by centrifugation at 30.000g, 4°C for 15 minutes. The cleared lysate was incubated 4 hours at 4°C on a roller with Ni-NTA Superflow resin (Qiagen), which had been washed 3 times with core buffer before use. The resin with the bound protein was loaded into an Econo-Pac Chromatography Column (BioRad) and the flow through was collected. The column was then washed 3 times with 20 ml core buffer with 20 mM imidazole. Proteins were eluted in 10 ml core buffer with 200 mM imidazole and 10 mM DTT and 1 ml fractions were collected. Purified protein was dialyzed against cold core buffer overnight at 4°C in a Slide-A-Lyzer Dialysis Cassettes, 3.5K MWCO (Thermo Fisher Scientific).

For 2xStrep-Src production and purification, Drosophila S2 Src cells were grown with 70 rpm agitation in a one-liter culture at 28°C until cell density reached 6x10^6^. Protein production was induced with 4 μM CdCl_2_ for 5 days. Cells were pelleted at 4.000g, 4°C for 15 minutes, resuspended in 25 ml buffer A1 (10 mM Tris pH 8, 150 mM NaCl, 1 mM EDTA) and lysed in a high-pressure homogenizer at 1.3 KBar, 4°C. Strep-PLCγ1 cSH2 was produced as described for GST-fused proteins, but the pellets were resuspended in 50 ml buffer A1 and lysed in a high-pressure homogenizer at 1.5 KBar, 4°C. After clearing the lysates at 75.000g, 4°C for 30 minutes, supernatants were filtered (0.22 μm) and applied to an 8 ml Strep-Tactin column on an ÄKTA FPLC at a flow rate 2 ml/min with buffer A1. 30 ml of B1 buffer containing 10 mM Tris pH 8, 150 mM NaCl, 1 mM EDTA and 2.5 mM Desthibiotin were used for the elution. Protein containing fractions were pooled together, concentrated and subjected to a gel filtration with GF buffer (100 mM Tris pH 7.5, 100 mM NaCl).

### Cell culture, transfection, transduction

HEK 293 (ATCC, CRL-1573) and HEK 293T (DSMZ No. ACC 305) were maintained in DMEM (Gibco) with 10% heat-inactivated FBS (PAN Biotech). Cell lines stably infected with KSHV cloned in a bacterial artificial chromosome (BAC) were described previously [28]. They were maintained in DMEM (Gibco) with 10% heat-inactivated FBS (PAN Biotech) and 150 μg/ml Hygromycin B in case of HEK 293 Bac36 WT and HEK 293 Bac36 ΔK15 or in EGM-2 MV (Lonza), 0.5 μg/ml doxycycline and 100 μg/ml Hygromycin B in case of HuAR2T Bac36 WT and HuAR2T Bac36 ΔK15. The KSHV lytic cycle in all adherent cells was induced by an addition of a reactivation cocktail (1.25 mM sodium butyrate with 3–5% (v/v) supernatant of SF9 cells infected with an RTA expressing baculovirus (gift from J. Vieira, University of Washington, Seattle, USA).

The Drosophila S2 Src cell line used for the purification of the recombinant Src kinase was established by co-transfecting the pT1204 plasmid encoding full length chicken Src with the pT371 plasmid encoding a puromycin resistant gene with Effectene Transfection Reagent (Qiagen) according to the manufacturer’s instructions. For this, 5x10^6^ cells were seeded in a T25 flask with 5 ml of Schneider’s medium (Gibco) and transfected after 24h with 2 μg of pT1204 Src and 0.1 μg of pT371. Resistant cells were selected in the presence of 8 μg/ml puromycin and medium was changed to Insect Xpress medium (Lonza) for protein production.

HEK-293T cells were transfected with Fugene 6 Transfection Reagent (Promega) according to the manufacturer’s instructions. 5x10^5^ cells were seeded in each well of a 6-well plate (or 2.5x10^5^ cells well of a 12-well plate). The next day Fugene was incubated at room temperature with OPTI-MEM for 5 minutes, added to DNA and incubated for another 15 minutes. For each 1 μg DNA, 3 μl Fugene were used.

### Western blot analysis and antibodies

For Western blot analysis of cellular lysates, cells were lysed in 80 μl 1xSDS sample buffer (62.5 mM Tris pH 6.8, 10% (v/v) glycerol, 2% (v/v) SDS, 50 mM DTT, 0.01% Bromophenol Blue) per well of a 12-well plate. Lysates were sonicated on ice and cleared in a table top centrifuge at 20.000g, 4°C for 10 minutes. Samples were analysed by SDS-PAGE with 4% polyacrylamide in the stacking gel and 10% or 12% in the separating gel. For the determination of protein size Precision Plus Protein All Blue Prestained Protein Standards from BioRad was used. After separation, proteins were transferred to a 0.45 μm nitrocellulose membrane (Amersham) in cold transfer buffer (25 mM Tris-Base, 250 mM glycine, 20% (v/v) methanol) at 350mA for 70 minutes. For GST-pulldown assays proteins were stained with Ponceau S for 5 minutes prior 1 hour blocking with PBS-T with 5% non-fat milk (Carl Roth) or TBS-T with 5% IgG-free albumin (Carl Roth). Membranes were incubated at 4°C overnight with primary antibodies. This was followed by 3 washing steps (10 minutes each) with PBS-T or TBS-T, a one hour incubation at room temperature with secondary HRP-conjugated antibodies, and another 3 washing steps.

Rabbit anti-GAPDH (14C10), mouse anti-phospho-tyrosine (9411), rabbit anti-PLCγ1 (2822), rabbit anti-phospho PLCγ1 Y^783^ (2821), rabbit anti S-tag (8476) and rabbit anti-Src (2108) were purchased from Cell Signaling Technology. Mouse anti-beta-actin (A5441), rabbit anti-DSCR1 (D6694) were purchased from Sigma-Aldrich and mouse anti-ORF45 (SC-53883), mouse anti-k-bZIP (SC-69797) from Santa Cruz. Mouse anti-GST (640802) was purchased from Biolegend. The production of the rat anti-K15 monoclonal antibody was described previously [29]. The following secondary HRP-conjugated antibodies were used: rabbit anti-mouse IgG (Dako-Cytomation, P 0260), goat anti-rabbit IgG (Dako-Cytomation, P 0448), goat anti-rat IgG (Southern Biotech, 3050–05).

### Immunoprecipitation of phosphorylated proteins

HEK293 were plated at a density 5x10^5^ cells per well of a 6 well plate (2 wells were used per condition) and transfected the next day with K15-encoding or empty PFJ vector using Fugene. HEK293 BAC36 WT/ΔK15 cells were plated at density 3x10^6^ per T75 flask and HuART BAC36 at density 5.8x10^6^ cells per 15 cm culture dish. After 24 hours virus-infected cells were reactivated and 48 hours after reactivation washed with PBS and lysed in 600 μl (for HEK293) or 500 μl (for HuART) Tris buffer (50 mM Tris pH 7.5, 150 mM NaCl, 1% (v/v) NP-40, protease and phosphatase inhibitors). For the transfected cells 300 μl Tris buffer was used per well of a 6-well plate (48 hours after transfection). Lysates were incubated on ice for 30 minutes and cleared by centrifugation for 10 minutes at 20.000g, 4°C. Phosphotyrosine affinity beads (30 μl per IP) (Cytoskeleton, Inc.) were washed twice in 1 ml of PBST and centrifuged at 800g, 4°C for 1 minute. The lysates (1.8 mg per IP) were incubated overnight at 4°C, washed 3 times with 1 ml Tris buffer and the bound proteins were eluted for 5 minutes at room temperature in 30 μl 2x non-reducing SDS sample buffer (125 mM Tris pH6.8, 20% (v/v) glycerol, 4% (v/v) SDS, 0.005% Bromphenol Blue). The samples were centrifuged at 20.000g for 1 minute, supernatants were incubated for 5 minutes at room temperature with 1μl β-mercaptoethanol and analysed by western blot.

For co-immunoprecipitation, 1 μg of pFJ K15P and 1 μg of pTriex-4 PLCγ1 SA were co-expressed in HEK-293T cells. Thirty-two hours after transfection cells were washed once with PBS and lysed in 300 μl IP buffer per well of a 6-well plate. Lysates (500 μl) were incubated overnight at 4°C with 10μl Red Anti-Flag M2 beads (Sigma Aldrich), which were washed before 3 times with 300 μl IP buffer. After that, beads were subjected to 6 washing steps with 700μl IP buffer, bound proteins were eluted in 15 μl 5x Laemmli sample buffer and analysed by Western blot.

### In vitro kinase assays

The GST-fused cytoplasmic tail of pK15 (GST-pK15 CT) was *in vitro* phosphorylated by recombinant human GST-6xHis Src kinase (ActiveMotif, 0.4 μg/ml per kinase reaction, 10 minutes at 30°C), or a recombinant chicken 2xStrep-Src kinase (10 μg/ml per kinase reaction, 30 minutes at 25°C). GST-pK15 CT was used at 4.8 μM or 6 μM and 50 μl or 100 μl reaction volumes for AlphaLISA and 3–6 μl glutathione beads with K15 and 25 μl reaction volume for GST pulldown experiments. The kinase buffer for GST-6xHis Src was 60 mM HEPES pH7.5, 1.2 mM DTT, 3 mM MgCl_2_, 3 mM MnCl_2_, 0.2 mM ATP, 3 μM Na-orthovanadate, whereas for 2xStrep-Src 20 mM Tris pH7.4, 20 mM NaCl, 1 mM DTT, 10 mM MgCl_2_, 0.2 mM ATP, 0.5 mM Na-orthovanadate, 0.5 mM β-glycerophosphate were used.

To test the impact of a pK15 CT fragment (pK15 CT^N2^) or phosphorylated pK15 peptides representing the last 12 (DDLpYEEVLFPRN) or 35 (SILRVDGGSAFRIDTAQAATQPTDDLpYEEVLFRN) amino acids of pK15 CT on the Src-mediated phosphorylation of the PLCγ1 tSH2 domain, GST-pK15CT^N2^ was first phosphorylated with Src in vitro as described in the preceding paragraph. Phosphorylated GST-pK15 CT^N2^ or the phosphorylated pK15 peptides, suspended in 50 mM Tris pH7.4, 300 mM NaCl, 1 mM DTT, were then added to the purified recombinant tSH2 domain (0.6 μM) together with a limiting amount of recombinant Src kinase (0.085 μM) in kinase buffer (20 mM Tris pH7.4, 20 mM NaCl, 1 mM DTT, 10 mM MgCl2, 0.2 mM ATP, 0.5 mM Sodium orthovanadate, 0.5 mM β-glycerophosphate) and the reaction allowed to proceed for 45 min. The reaction mix was then analysed by Western blot using an antibody to the phosphorylated Y^783^ in the linker region of the PLCγ1 tSH2 domain and an antibody to the 5x His tag. To test the inhibitory effect of the phosphorylated 12mer pK15 peptide on the enhancement of PLCγ1 tSH2 domain phosphorylation induced by the pK15 35mer peptide, increasing concentrations (1.25–5.0 μM) of the 12mer peptide were added to a reaction mix containing the purified recombinant tSH2 domain (2.5 μM), the phosphorylated pK15 35mer (2.5 μM), and recombinant Src kinase (0.085 μM). Following a 45 min incubation at 37°C, the reaction mixture was analysed by Western blot as above.

### GST-pulldown

HEK293T cells were washed once with cold PBS 30 hours after transfection, lysed in IP buffer (50 mM Tris pH7.6, 60 mM NaCl, 6.25 mM EDTA, 1% (v/v) glycerol, 0.04% (v/v) NP-40) on ice for 30 minutes, sonicated shortly and cleared by centrifugation for 10 minutes at 20.000g, 4°C. The IP buffer also contained protease inhibitors: phenylmethylsulfonyl fluoride (PMSF) 100 μM, aprotinin 1.5 μM, benzamidine 1 mM, leupeptin 10 μM, pepstatin 1.46 μM. GST-fused proteins bound to glutathione beads were incubated overnight at 4°C upon agitation with 300 μl cell lysates in the presence of phosphatase inhibitors (PhosStop, Roche) if necessary. Alternatively, GST-fused proteins bound to glutathione beads were incubated with 5 μg of the purified His-tagged protein in the presence of 10 μg BSA (New England Biolabs) in IP buffer (300 μl final volume). Beads were then washed 5 times in 500 μl IP buffer (or for purified proteins IP buffer with 200 mM NaCl), suspended in 10 μl of 5x Laemmli sample buffer (60 mM Tris pH6.8, 24% (v/v) glycerol, 2% (v/v) SDS, 5% (v/v) β-mercaptoethanol, 0.01% Bromphenol Blue) and analysed by western blot.

### AlphaLisa

His-tagged PLCγ1 (SA, tSH2 or the isolated SH2 domains) was incubated at room temperature with GST-tagged K15P or its mutants for 1 hour. Both proteins were diluted in Alpha buffer (1xPBS, 0.5% BSA, 0.01% Tween 20) and if indicated, pK15 proteins were phosphorylated by Src kinase before use in the AlphaLisa. Ni chelate acceptor beads and glutathione donor beads (PerkinElmer) were diluted in the Alpha buffer and added sequentially to the proteins for another hour of incubation at room temperature. All manipulations with Alpha beads were performed under subdued light conditions. Final concentration of each protein was 300 nM and of each bead type 4 μg/ml in 25 μl total Alpha reaction volume in the well of an OptiPlate-384 (PerkinElmer). Emission at 615 nm was detected on BioTek Synergy 2 plate reader.

### Measuring the affinity of a pK15 peptide for PLCγ1 domains

The 12mer pK15 peptide (DDL(pY)EEVLFPRN) was purchased pre-labelled with 5,6-FAM at the N-terminus from Caslo ApS (Lyngby, Denmark) and was dissolved in buffer (100 mM MES, 150 mM NaCl, 3 mM TCEP (Tris(2-carboxyethyl)phosphin, ROTH), pH 6.5) and diluted to a final concentration of 200 nM for the experiments. To measure its affinity for PLCγ1 domains (tSH2, tSH2 R^586^L mutant, tSH2 R^694^L mutant, nSH2, nSH2 R^586^L mutant, cSH2, cSH2R^694^L mutant) in a microdiffusion chamber the peptide concentration was held constant at 200 nM in each sample, while the concentration of the PLCγ1 domains was varied from 0 nM to 8000 nM. The nSH2, cSH2 and tSH2 domains comprised residues 545–662, 668–790 and 545–790, respectively. All samples were prepared at the same time, equilibrated at room temperature for 30 minutes to reach the steady state and then tested in order, from the highest to the lowest protein content. A 5 μL aliquot of each sample was pipetted onto a microfluidic chip and tested using a Fluidity One-W instrument. Each sample was tested in triplicate.

The values of the hydrodynamic radius R_h_ of the pK15–protein complex in each solution were generated by the Fluidity One-W software; the average values were fitted to a standard binding equation. *K*_D_ values were determined by non-linear least squares fitting of the data with Equation 1 [71].

$$y=R_{h,free}+{(R}_{h,complex}-R_{h,free})\frac{(K_{D}+A+nx)-\sqrt{{\left(K_{D}+A+nx\right)}^{2}-4nAx}}{2nA}$$

where *y* is the hydrodynamic radius of the mixture measured on Fluidity One-W, *R*_*h*,*free*_ is the hydrodynamic radius of free peptide, *R*_*h*,*complex*_ is the hydrodynamic radius of the peptide bound to the PLCγ1 domain, *x* is the concentration of the PLCγ1 domain, *K*_D_ is the dissociation constant of the complex, *n* is the number of binding sites of the PLCγ1 domain and A is the concentration of the peptide.

### NMR spectroscopy

NMR experiments were recorded at 298 K on a 600-MHz Bruker Avance III-HD spectrometer equipped with an inverse HCN cryogenic probehead (nitrogen-cooled) and running Topspin 3.2 software. 2D ^15^N-HSQC spectra were recorded using States-TPPI for frequency discrimination, with water suppression achieved via a combination of WATERGATE and water flip-back pulses to preserve the water magnetization [72,73]. NMR data were processed in Topspin and analysed in CcpNmr Analysis v2.4 [74].

### Structure of the PLCγ1 tSH2 domain in complex with a phosphorylated pK15 peptide

A shortened version of the PLCγ1 tSH2 domain (aa 545–772) lacking the linker peptide connecting the PLCγ1 cSH2 and SH3 domains (see Results section) was cloned into a pET28a vector carrying an N-terminal His Tag and a SUMO sequence. Expression was carried out in *E*. *coli* and the protein was purified to homogeneity as described above. His-Tag and SUMO were proteolytically removed using SUMO protease (SIGMA) following the manufacturer’s instructions, and the cleaved protein purified on a HisTrap column. Fractions containing cleaved protein were further purified by size exclusion chromatography (SEC) using a Superdex 75 26/600 column (GE Healthcare) equilibrated with 50mM Tris pH7.5, 150mM NaCl and 1mM TCEP buffer. The purified protein in SEC buffer was added to lyophilized phosphorylated 12mer pK15 peptide (DDLpYEEVLFPRN) in a 1:8 molar ratio (Protein: peptide) at a final concentration of 3mg/ml of peptide and 7.5mg/ml protein. Crystals were grown at 293 K using the sitting-drop vapor diffusion method in drops containing 1μl protein solution mixed with 1 μL reservoir solution containing 30% PEG 4000, 0.1M TrisHCl pH 8.5 and 0.2M sodium acetate. The crystals were flash frozen in the mother liquor containing 30% ethylene glycol and diffraction data were collected at P13 at DESY-Hamburg. Data were processed, scaled and reduced with XDS [75], Pointless [76] and programs from the CCP4 suite [77]. The phase problem was overcome by the molecular replacement method using 4FBN [63] as search model in Phaser [78]. Model building was performed using Coot [79], and refinement was done using AutoBuster [80] with repeated validation using MolProbity [81]. Figures were generated using PYMOL (http://www.pymol.org/2/support.html).

## Supporting information

S1 Fig Both pK15 Y^431^ and Y^481^ are phosphorylated by recombinant Src kinase. GST-fused pK15 CT (WT), the pK15 CT Y^481^F mutant (Y^481^F), the pK15 CT Y^431^F mutant (Y^431^F), or GST were bound to glutathione beads and phosphorylated by GST-6xHis Src (+) or left unphosphorylated (-). Proteins were then analysed by WB using an antibody to pTyr.
(TIF)
