# Supplementary figures and images for: Recruitment of phospholipase Cγ1 to the non-structural membrane protein pK15 of Kaposi Sarcoma-associated herpesvirus promotes its Src-dependent phosphorylation
Source: PLoS Pathog. 2021 Jun 18;17(6):e1009635. doi: 10.1371/journal.ppat.1009635 (PMC8244865; doi:10.1371/journal.ppat.1009635)

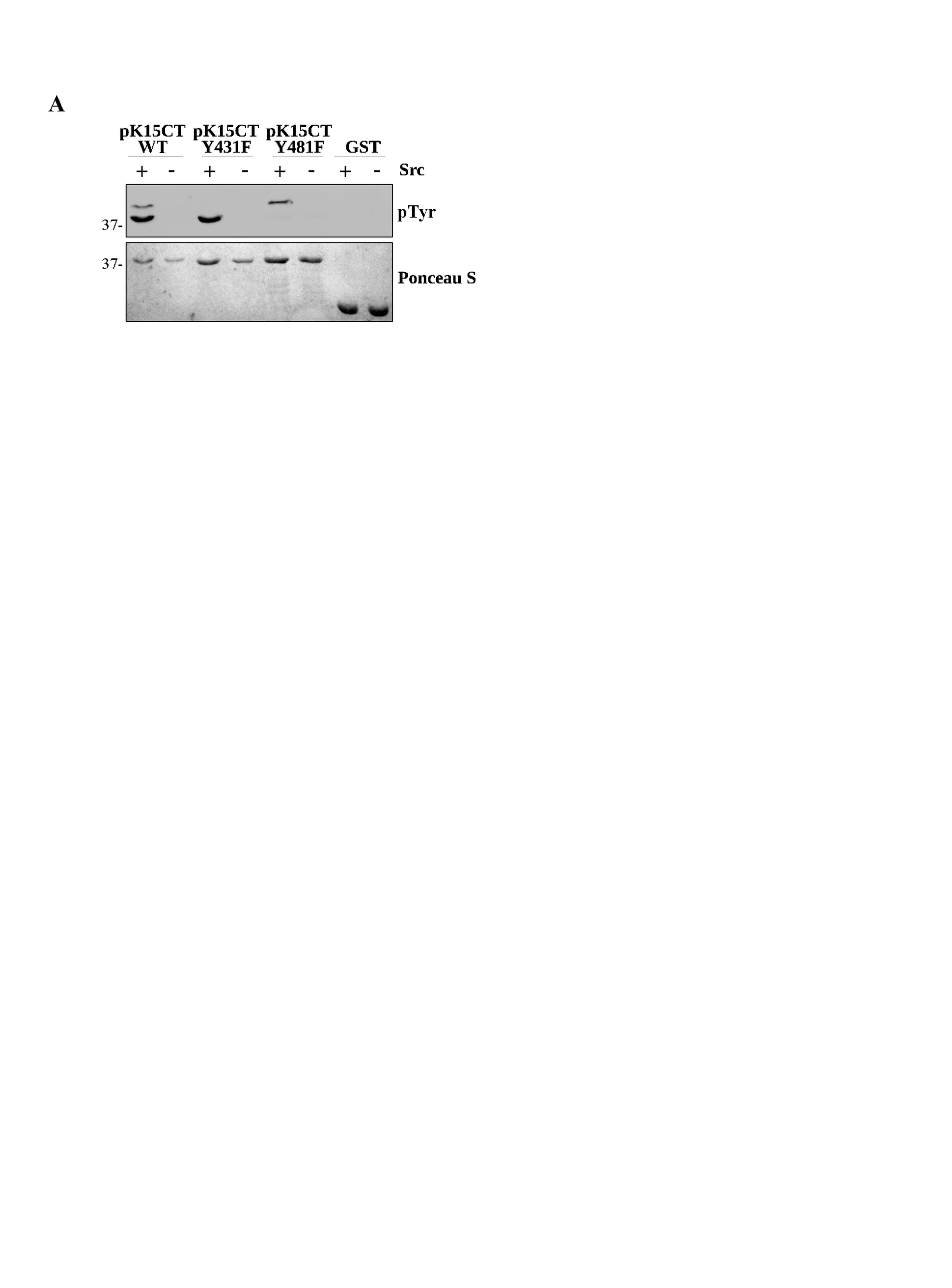

Supplement: S1 Fig — GST-fused pK15 CT (WT), the pK15 CT Y481F mutant (Y481F), the pK15 CT Y431F mutant (Y431F), or GST were bound to glutathione beads and phosphorylated by GST-6xHis Src (+) or left unphosphorylated (-). Proteins were then analysed by WB using an antibody to pTyr. (TIF) [file ppat.1009635.s001.tif]
